# *Cachrys* L. Genus: A Comprehensive Review on Botany, Phytochemistry and Biological Properties

**DOI:** 10.3390/plants12030565

**Published:** 2023-01-26

**Authors:** Vincenzo Musolino, Maria Rosaria Perri, Filomena Conforti, Micaela Gliozzi, Mariangela Marrelli, Vincenzo Mollace

**Affiliations:** 1 Laboratory of Pharmaceutical Biology, Department of Health Sciences, Institute of Research for Food Safety & Health (IRC-FSH), University Magna Graecia of Catanzaro, 88100 Catanzaro, Italy; 2 Department of Pharmacy, Health and Nutritional Sciences, University of Calabria, 87036 Rende, Italy; 3 Department of Health Sciences, Institute of Research for Food Safety & Health (IRC-FSH), University Magna Graecia of Catanzaro, 88100 Catanzaro, Italy

**Keywords:** Apiaceae, bioactive compounds, *Cachrys*, coumarins, essential oils, ethnobotany, phytochemicals, photocytotoxicity

## Abstract

The *Cachrys* L. genus belongs to the Apiaceae family and it is widely distributed in the Mediterranean basin, with plant species being endemic to southern Europe, Asia, and northern Africa. Different studies, focused on the phytochemical composition of *Cachrys* spp. and the biological properties of their phytocomplexes, have been reported. These works mostly focused on the essential oils obtained from these plants, and pointed out that *Cachrys* species are a rich source of coumarins, mainly furanocoumarins. Other phytochemicals, such as terpenes, fatty acids, phytosterols, and flavonoids have been also identified. Moreover, a number of biological properties such as antioxidant, antimicrobial, anti-inflammatory, cytotoxic, and photocytotoxic effects have been assessed. Nevertheless, a review of the chemical and pharmacological properties of this genus is not available in the literature. The aim of this paper is to provide an overview of the reports concerning the identified phytochemicals and the biological effects reported for *Cachrys* spp., and to offer a comprehensive understanding of the potential of this genus as a source of bioactive compounds. The current taxonomy, the traditional uses, and the toxicological aspects of plants belonging to this genus are also reported, and the future research directions are discussed.

## 1. Introduction

*Cachrys* L. is an accepted genus belonging to the Apiaceae family, native to Mediterranean countries and distributed in southern Europe, northern Africa, and Asia. The species belonging to this genus are present in Turkey, Albania, Algeria, Bulgaria, Iran, Greece, Italy, Krym, Libya, Morocco, North Caucasus, Portugal, Romania, South European Russia, Spain, Transcaucasus, and Tunisia [1,2].

The *Cachrys* genus contains 15 known species, with 11 accepted species including *C. aksekiensis* (A.Duran & B.Doğan) Hand, *C. alpina* M.Bieb., *C. boissieri* (Reut. & Hausskn. ex Boiss.) Hand, *C. caspica* (DC.) Menitsky, *C. crassiloba* (Boiss.) Meikle, *C. cristata* DC., *C. libanotis* L*., C. microcarpos* M.Bieb., *C. pungens* Jan ex Guss, *C. scabra* (Fenzl) Meikle, and *C. sicula* L. [3].

Coumarins and furanocoumarins have been identified as the main secondary metabolites of these species, with bergapten, xanthotoxin, and isopimpinellin being the most abundant molecules [4,5,6]. Different studies also described the volatile metabolites of *Cachrys* spp. [7,8].

A survey of the literature allowed us to highlight a number of biological properties exerted by these plants, including antioxidant, anti-inflammatory, antimicrobial, cytotoxic, and photocytotoxic effects, as well as some enzyme inhibitory properties [5,9].

Overall, beside the difficult taxonomy which characterizes this genus, and the somewhat confused classification of *Cachrys* species, this genus appears to be strongly under-investigated, both from a chemical and a biological point of view. Some of the species belonging to this genus have not yet been explored, and a limited number of studies have been reported till now compared to many other plant genera. 

Although these studies have illustrated such interesting chemical and biological properties, they have not been reviewed so far. The aim of this review is to provide a comprehensive summary of the botanical and ethnobotanical information, the phytochemical composition, the biological properties, and the toxicological aspects of plants belonging to the *Cachrys* genus. The state of the art is presented in order to provide a comprehensive understanding of the prospective of this genus as a source of bioactive compounds.

## 2. Methodology

A review of the literature was performed in accordance with the Preferred Reporting Items for Systematic Reviews and Meta-Analyses (PRISMA) guidelines [10]. This search comprises references published until November 2022. Google Scholar, Scopus, and Pubmed search engines were used, and the following keywords have been searched: “*Cachrys*” and the scientific binomial of each synonym “*Bilacunaria aksekiensis*”, “*Hippomarathrum boissieri*”, “*Bilacunaria caspia*”, “*Echinophora capsica*”, “*Hippomarathrum caspicum*”, “*Cachrys echinophora*”, “*Hippomarathrum cristatum*”, “*Hippomarathrum pauciradiatum*”, “*Trachymarathrum siculum*”, “*Cachrydium libanotis*”, “*Cachrys humilis*”, “*Cachrys linearia*”, “*Cachrys peucedanoides*”, “*Cachrys pterochlaena*”, “*Cachrys sphaerocarpa*”, “*Crithmum libanotis*”, “*Hippomarathrum bocconei*”, “*Hippomarathrum libanotis*”, “*Lophocachrys echinophora*”, “*Smyrnium libanotis*”, “*Bilacunaria macrocarpa*”, “*Cachrys amplifolia*”, “*Cachrys crispa*”, “*Cachrys longiloba*”, “*Cachrys macrocarpa*”, “*Cachrys nudicaulis*”, “*Hippomarathrum amplifolium*”, “*Hippomarathrum crispum*”, “*Hippomarathrum longilobum*”, “*Hippomarathrum microcarpum*”, “*Hippomarathrum pungens*”, “*Bilacunaria scabra*”, “*Ferula scabra*”, “*Ferulago scabra*”, “*Hippomarathrum scabrum*”, “*Cachrys crispata*”, “*Cachrys pterochlaena*”, “*Hippomarathrum brachylobum*”, “*Hippomarathrum siculum*”, “*Seseli brachylobum*”, “*Smyrnium hispidum*”, “*Ulospermum siculum*”.

The World Flora Online (WFO) (http://www.worldfloraonline.org/, accessed on 28 November 2022) and Plants of the World Online (https://powo.science.kew.org/, accessed on 28 November 2022) databases were used to validate the scientific names [3,11]. 

Overall, 238 references were identified, of which 165 papers were found on Google Scholar, 52 on Scopus, and a further 21 on Pubmed. Duplicates (74 papers) were removed, and the remaining literature was selected based on the titles and abstracts, and on the basis of the following inclusion criteria: studies published in English, articles whose title and/or abstract referred to *Cachrys* species, articles whose title contained synonyms of *Cachrys* species. After this primary screening, 101 manuscripts were excluded and two full-papers were not retrieved. The remaining 61 full-text records were thoroughly inspected. Finally, 34 references were included in this review.

**Figure 1:**
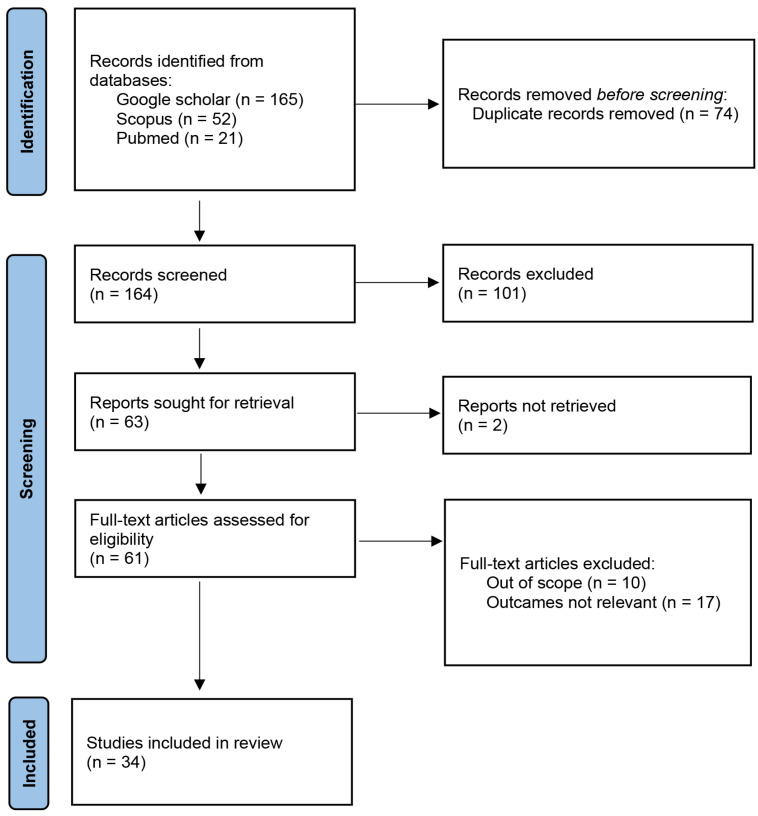
Selection process of the bibliographic sources based on a PRISMA 2020 flow diagram.

The selection process of the bibliographic sources is described in Figure 1.

The 34 studies included in this report were from authors from different countries. It is noticeable that the majority of them are from Turkey (33%) and Italy (27%), followed by Spain (9%), Iran (9%), Serbia (8%), and Algeria (7%). Turkey has the highest Apiaceae diversity in Asian countries and probably in the world, with about 150 species in 42 genera [12,13,14].

**Figure 2:**
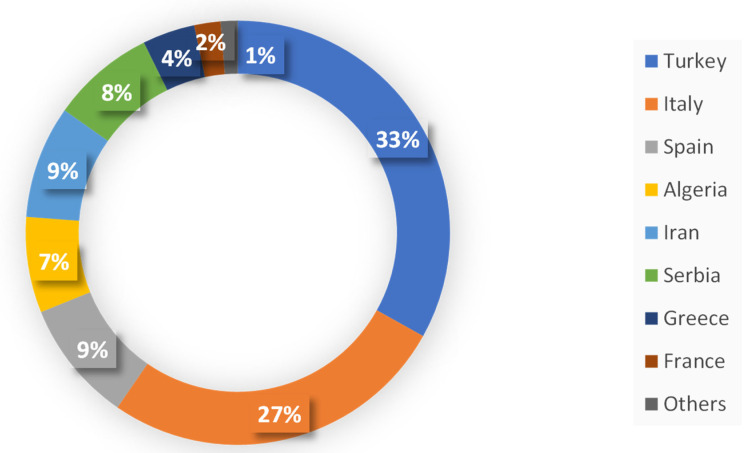
Distribution of authors by country of origin.

Figure 2 reports the distribution of authors by their country of origin.

**Figure 3:**
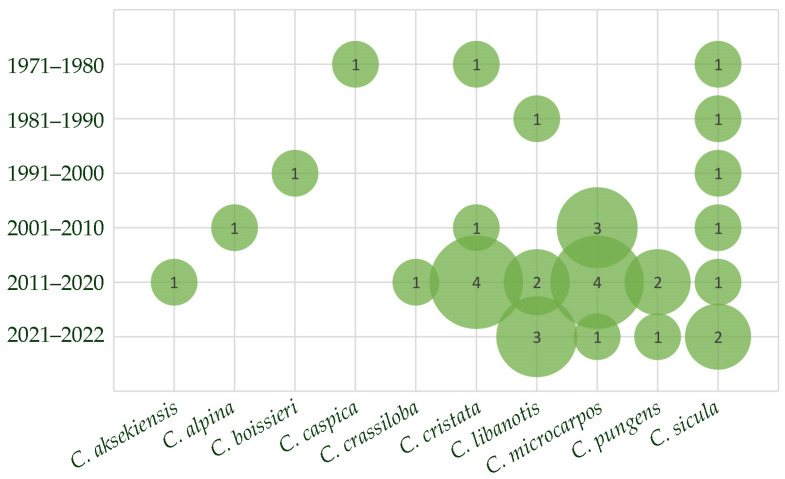
Bubble chart showing the number of papers focused on each *Cachrys* species in a specific period.

Figure 3 presents a bubble chart showing the number of papers dealing with the different *Cachrys* species, and the period of publication. As shown, the interest towards these species is increasing in the last years, with *C. microcarpos* M.Bieb., *C. sicula* L., *C. cristata* DC., and *C. libanotis* L. being the most investigated plants.

## 3. Taxonomy of the *Cachrys* Genus

The classification of the *Cachrys* genus has been somewhat confused. According to Tutin (1968), this genus included also *Prangos* Lindley and *Hippomarathrum* Link, while in 1872, Boissier had classified them into three different genera [15,16,17].

Currently, according to The World Flora Online (WFO) database (http://www.worldfloraonline.org/, accessed on 28 November 2022), the *Cachrys* L. genus belongs to the Apiaceae family, and it contains 15 known species, with 11 accepted species [3]. 

Apiaceae is a large family with a complex taxonomy, which comprises 430 genera and about 3780 species, most of which belong to the subfamily Apioideae [18].

The *Cachrys* genus is present from Mediterranean countries to Iran: Albania, Algeria, Bulgaria, Cyprus, East Aegean Is., Greece, Iran, Italy, Krym, Libya, Morocco, North Caucasus, Portugal, Romania, South European Russia, Spain, Transcaucasus, Tunisia, Turkey [1,2].

According to the molecular phylogenetic studies by Mousavi and colleagues, the genera *Azilia*, *Bilacunaria* Pimenov & V.N.Tikhom., *Cachrys* L., *Diplotaenia* Boiss., Eriocycla, *Ferulago* W.D.J.Koch, and *Prangos* belong to the *Cachrys* clade, and are all widely distributed from southern Europe, northern Africa, Turkey, central and Southwest Asia, to China [18].

Table 1 reports the included species and the taxonomic synonyms. 

## 4. Botanical Description and Ethnobotanical Uses

*Cachrys* species are described as much-branched, robust perennial herbs with deeply dissected leaves. They have compound umbels and hermaphrodite flowers. The calyx has five short and distinct teeth, and petals are obovate, usually yellow. The fruit is ovoid to subglobose, crowned with a broad disk [1,19].

Unlike other genera, whose plants have been widely empirically used in popular medicine for their beneficial effects, there is not much information in the literature about the traditional uses of *Cachrys* species.

*Cachrys libanotis* has been reported to be traditionally used in the treatment of rheumatism [20]. *Cachrys alpine* M.Bieb. is instead used as an aphrodisiac in Balikesir province in Western Turkey, where it is known as ‘Çaksir otu’ [21]. 

In Eastern Anatolia (Turkey) many wild edible plants are traditionally used in daily diets. These plant species are commonly used as fresh vegetables, dried herbs, or as pickled plants in some sole or mixed milk and meat products [22]. *C. cristata* DC. (*Hippomarathrum cristatum* Boiss.) is also traditionally used in Turkey, where it is known as “Tarhana otu” or “Çaşır”, and is added to a traditional tarhana soup [23].

Moreover, in the Eastern Anatolia region of Turkey, *C. microcarpos* M.Bieb. is still used by the farmers as an alternative source of roughage. Even if there are few studies in the literature concerning the use of the *H. microcarpum* plant as a source of coarse feed in animal feeding, the nutritional content of this plant has been reported by Gül [24].

## 5. Phytochemical Constituents

Phytochemical studies on the *Cachrys* species allowed the characterization of coumarins, terpenoids, phenylpropanoids, phenolic compounds, fatty acids, and phytosterols. Overall, the phytochemical investigations carried out till now allowed the identification of 177 secondary metabolites. The remarkable furanocoumarin content which characterizes *Cachrys* species is particularly interesting, given the growing importance that these compounds are assuming in dermatology and oncology. Indeed, furanocoumarins are useful photosensitizing agents in photodynamic therapy. Most of the studies concerned the phytochemical characterization of essential oils from *Cachrys* species, but in some cases, extracts obtained through maceration or Naviglio**^®^** extractor have also been analyzed. 

### 5.1. Coumarins

Coumarins, mainly furanocoumarins, stand out among the main phytochemical compounds identified in *Cachrys* species.

Coumarins (1,2-benzopyrone or 2H-1-benzopyran-2-one) and their derivatives are natural compounds widely found both as a heteroside and free form in the seeds, roots, and leaves of many plant species, mainly belonging to the Apiaceae and Rutaceae families [25,26]. Furanocoumarins are tricyclic aromatic compounds bearing a furan ring fused to the α-benzopyrone (coumarin) system. These derivatives can be structurally divided into angular and linear furanocoumarins, with the furan ring attached at the 6,7 positions on the aromatic ring (e.g., angelicin) or to the 7,8 positions (e.g., psoralen), respectively [27].

In 1973, Abishev and coworkers reported the presence of several coumarins from the roots of *Hippomarathrum capsicum* (DC.) Grossh., actually a synonym of *C. caspica* (DC.) Menitsky. Osthole, isoimperatorin, oxypeucedanin, umbelliferone, heraclenin, and pangelin were isolated from the chloroform root extract [28]. 

In 1979, Doganca and colleagues isolated the coumarin ulopterol from *Hippomaathrum cristatum* (a synonym of *C. cristata* DC.) [29].

In 1986, Grande and colleagues described the presence of some coumarins in benzene extracts from *C. sicula*. Isoimperatorin, imperatorin, bergapten, xanthotoxin, isopimpinellin, *tert*-*o*-methylheraclenol, prantschimgin, sprengelianin, and ulopterol were identified in the umbels with NMR and IR analyses, and mass spectrometry (MS), while the furanocoumarins imperatorin, salaxin, pabulenol, oxypeucedanin, prantschimgin, and sprengelianin were detected in the root extract [4]. 

Marrelli and colleagues investigated the phytochemical profile of *Cachrys sicula* and *Cachrys libanotis* extracts obtained through two different extraction techniques, a traditional solvent maceration and a pressurized cyclic solid-liquid extraction technique, the Naviglio extraction^®^. The GC-MS qualitative profile revealed the presence of three furanocoumarins: xanthotoxin, bergapten and isopimpinellin, in each investigated extract. Xanthotoxin was identified in the highest amount in both the extracts obtained with Naviglio^®^, with percentages equal to 14.8% and 10.8% for *C. libanotis* and *C. sicula*, respectively. Bergapten, another naturally occurring furanocoumarin, was detected with percentages from 1.8% to 2.8%. The same trend was observed as regards isopimpinellin. A quantitative analysis was also carried out. Consistent with previous analyzed data, *Cachrys libanotis* and *Cachrys sicula* extracts, both obtained through the innovative Naviglio extraction^®^ technique, showed the highest amount of xanthotoxin, with values equal to 4.98 ± 0.21 and 4.10 ± 0.23 mg/mL, respectively. As regards bergapten, the same trend can be observed, *Cachrys libanotis* extract obtained with the Naviglio^®^ extractor being the richest sample both in bergapten and xanthotoxin. On the contrary, isopimpinellin showed the highest value in the *Cachrys sicula* extract obtained through the Naviglio^®^ instrument. Seselin (a pyranocoumarin), as well as osthol, suberosin, and isogeijerin (coumarins), were found in *Cachrys libanotis* macerated extract, while the pyranocoumarin 2-methyl-2-butenoic acid 9,10-dihydro-8,8-dimethyl-2-oxo-2H,8Hbenzo[1,2-b:3,4-b0] dipyran-9-yl ester was recognized in *Cachrys sicula* and *Cachrys libanotis* extracts obtained through the Naviglio^®^ extractor at 10.2% and 9.7%, respectively [5].

The *Cachrys pungens* extract obtained through Naviglio^®^ extraction was also standardized in furanocoumarins content through GC-MS quantitative analyses by using bergapten as an external standard. The results showed amounts of xanthotoxin, bergapten, and isopimpinellin equal to 7.48 ± 0.48, 2.94 ± 0.16 and 1.07 ± 0.14 mg/50 mg of extract, respectively [9]. Menichini and coworkers investigated the phytochemical composition of *Cachrys pungens* collected in Calabria, southern Italy. In the chloroform fraction, a total of 8 coumarins were identified: psoralen, 3-methylsuberosine, xanthotoxin, bergapten, isopimpinellin, isooxypeucedanin, jatamansin, and columbianetin [6].

De Leo and colleagues isolated for the first time the compound 8-hydroxymethylpsoralen, a new coumarin, together with the known sprengelianin and (RS)-oxypeucedanin in *Cachrys sicula* leaves extract [30].

### 5.2. Terpenoids

A huge number of terpenoids have been identified in species belonging to *Cachrys* genus. Due to the high content of this class of phytochemicals, a *Cachrys* species, namely *C. cristata* DC., was one of the 21 plant species from Greece investigated by Evergetis and coworkers for their potencies as essential oils (EOs) producing crops and as renewable sources for the production of fine chemicals. *C. cristata* resulted one of the most potent EO producing crops with an estimated production yields per hectare exceeding the 30 L, with the compound myrcene accounting for estimated 19 L [31]. 

Baser and coworkers described the phytochemical composition of the essential oil from the fruit of *C. alpine* M.Bieb from Turkey [21]. The authors reported that the microdistilled oil from the dried fruits of this species contained different terpenes, with α-humulene being the major constituent (33.1%). *p*-Cymene, α-phellandrene, and germacrene D were present in percentages above 8%, and α-pinene (6.3%) was also identified among the most abundant components. β-Pinene, β-phellandrene, β-caryophyllene, bicyclogermacrene, germacrene B, and other minor constituents were also identified. 

Bourderdara and colleagues investigated the phytochemical profile of *Cachrys libanotis* aerial parts’ essential oils. The composition, assessed through GC-MS analysis, indicated the presence of monoterpenes (31.9%) and sesquiterpenes (45.4%). Germacrene-D represented the main compound (18.0%), followed by γ-terpinene, *p*-cymene and limonene (6.4%, 5.5%, 5.1%, respectively) [7].

Palá-Paúl and coworkers described the terpene composition of the essential oils from *C. sicula* L. aerial parts. The essential oils were obtained from different parts of the plant: stems and leaves, umbels, fruits and flowers. Only quantitative differences were detected among the samples, but they were similar from a qualitative point of view, with *p*-cymene, γ-terpinene, α-pinene, sabinene, myrcene, and ocimene being the major constituents [15].

The phytochemical composition of a *C. sicula* essential oil was also reported by Tahar and coworkers, who analyzed the essential oil from the leaves of the plant. Thirty-two compounds, accounting for about 99% of the total oil, were identified, with β-pinene, sabinene, myrcene, and α-pinene being the main constituents [32]. 

The phytochemical profile of *Cachrys sicula* and *Cachrys libanotis* extracts, obtained through both maceration and Naviglio^®^ extractor, showed the ubiquitous presence of the terpene estragole, while limonene, fenchone, and anethole were only detected in the *Cachrys sicula* extract obtained with the Naviglio^®^ extractor [5].

The *n*-hexane fraction of the *Cachrys pungens* extract analyzed by Menichini and colleagues revealed the presence of 17 terpenes, divided into monoterpenes, sesquiterpenes, sesquiterpene alcohols, a diterpene, and a triterpene. Most of these molecules were present in traces, while β-phellandrene, carvacrol, trans-caryophyllene, β-farnesene, α-longipinene, and β-bisabolene were the most abundant ones [6].

The phytochemical profile of *Cachrys cristata* essential oils was investigated with GC-MS. This identified 48, 40, and 22 terpenes, in the leaf, stem, and root essential oils, respectively. These were classified into monoterpene hydrocarbons, oxygenated monoterpenes, sesquiterpene hydrocarbons, and oxygenated sesquiterpenes. (Z)-β-Ocimene, a monoterpene hydrocarbon, was the most abundant compound in each analyzed sample, accounting for 44.25%, 30.55%, and 15.2% in the leaf, stem, and root essential oils, respectively. The leaf essential oil was also rich in δ-3-carene, γ-terpinene, terpinolene, and β-caryophyllene (8.7%, 6.0%, 5.2%, and 5.5%, respectively). The stem essential oil was particularly rich in monoterpene hydrocarbons, with *p*-cymene, δ-3-carene, γ-terpinene, and terpinolene accounting for 10.7%, 8.8%, 6.9% and 6.7%, respectively. Among the other components, α-phellandrene (11.7%) was identified in the root essential oil [33].

Ozer and colleagues investigated the phytochemical composition of the leaf extract and essential oil of *C. microcarpos* M.Bieb. (syn. *Hippomarathrum microcarpum*). Bornyl acetate, caryophyllene oxide, β-caryophyllene, and pinocarvone, in percentages equal to 19.9%, 7.7%, 6.3%, and 4.2%, were the main identified terpenes in the essential oil [34]. Karakaya and coworkers reported the chemical composition of the essential oil from the aerial parts of the same species, *C. microcarpos* M.Bieb. (syn. *Hippomarathrum microcarpum*). They identified 21 compounds, with β-caryophyllene, caryophyllene oxide, bornyl acetate, humulene, germacrene-D, and α-phellandrene being the most abundant compounds, with percentages of 31.4%, 23.1%, 9.1%, 4.9%, 4.2%, and 4.6%, respectively [35].

The composition of the essential oil from this same species was also investigated by Khalilzadeh and colleagues, who assessed the phytochemical content of the essential oil from the aerial parts of *C. microcarpos* M.Bieb. (syn. *Hippomarathrum microcarpum*) obtained by hydrodistillation. The authors reported α-pinene, β-caryophyllene, β-phellandrene, and germacrene-D as the major components [8].

Furthermore, the chemical composition of *C. microcarpos* was also investigated by Sefidkon and Shaabani, who described the phytochemical content of the essential oil from the leaves and flowers of the plant. β-Caryophyllene, y-muurolene, and linalool were found to be the major components of the leaf essential oil. These same compounds, together with thymol and camphene, were also the main constituents of the essential oil obtained from the flowers [36]. 

Özek and coworkers verified the phytochemical composition of the essential oil from the fruits of this same *Cachrys* species. The plant material from *C. cristata* was subjected to hydrodistillation, and the analysis were carried out with GC-MS, with sesquiterpenes resulting as the most abundant class of terpenes (accounting for about 38% of the oil), and germacrene D and germacrene B being the major identified terpenoids [23].

The phytochemical composition of *C. boissieri* (Reut. & Hausskn. ex Boiss.) Hand (syn. *Hippomarathrum boissieri*) was also investigated. Baser and coworkers reported the terpenoids identified in the essential oil from the aerial parts of this species, which were distilled using a Clevenger-type apparatus. GC-MS analyses revealed the presence of eighty-nine compounds, with β-caryophyllene, caryophyllene oxide, and α-pinene (8.8%) being the major compounds [37].

### 5.3. Phenylpropanoids

The presence of some phenylpropanoids (4.9%) was reported for *C. cristata*. Myristicin was found to be the major component belonging to this class of compounds in the essential oil obtained from the fruit of this species, followed by methyl eugenol [23].

The phenylpropanoid eugenol has been also reported in the essential oil from the aerial parts of *C. boissieri* [37].

### 5.4. Phenolic Compounds

Few data on the phenolic composition of *Cachrys* spp. have been reported. The ethyl acetate fraction of *C. pungens* aerial parts in methanolic extract was explored using high-performance thin-layer chromatography (HPTLC), which allowed identification of the flavonoid catechin, the flavonoid glycosides naringin and quercitrin, the cinnamic acids caffeic and ferulic acids, and also gallic acid, a phenolic acid [38].

### 5.5. Fatty Acids

The presence of some fatty acids has been reported for *C. sicula* and *C. libanotis*. Palmitic acid was identified in both *Cachrys* species, while myristic acid and α-linoleic acid were identified in percentages equal to 0.2% and 0.7% in *Cachrys libanotis* extracts obtained through Naviglio^®^ extraction and traditional solvent maceration, respectively [5].

The *n*-hexane fraction of *C. pungens* extract obtained through maceration revealed the presence of hexadecanoic acid (palmitic acid) and 8,11-octadecadienoic acid methyl ester in percentages equal to 2.5% and 2.2%, respectively, together with some other minor constituents belonging to this class, among which was linoleic acid (9,12-octadecadienoic acid) [6]. 

The fatty acid palmitic acid was also detected in the aerial parts of *C. microcarpos* M.Bieb. (syn. *Hippomarathrum microcarpum*) [8].

Küçükboyacı and coworkers carried out an interesting study aimed to assess the fatty acid composition of the seed oil from a number of Apiaceae species from Turkey, including *C. cristata* DC. (*Hippomarathrum cristatum* Boiss.) and *C. microcarpos* (*Hippomarathrum microcarpum*). In particular, the purpose of the authors was to verify the content of petroselinic acid, a valuable oleochemical raw material whose double bond can be oxidatively split to form lauric and adipic acids, two fatty acid used for the production of surfactants and soaps. Petroselinic acid was found to be the major fatty acid present in the seed oils of *C. cristata* (72.2%). Linoleic acid was instead the most abundant component of *C. microcarpos* seed oil (36.9%) [13]. 

Özek and coworkers verified the phytochemical composition of the essential oil from the fruits of *C. cristata*, reporting the presence of hexadecanoic acid (palmitic acid), accounting for 11.6% of the essential oil, together with other fatty acid methyl and ethyl esters [23].

### 5.6. Phytosterols and Others

According to Menichini and coworkers, six phytosterols were identified in the *n*-hexane fraction of *C. pungens* extract, with stigmasta-5,22-dien-3-ol and γ-sitosterol being the most abundant ones. The other recognized molecules were campesterol, 9,19-cyclolanostan-3-ol,24-methylene, cycloartenol, and stigmast-7-en-3-ol [6].

Moreover, Pinar and Alemany reported the isolation of N-N’-di-*o*-tolylethylendiamine from *C. sicula*. This molecule was identified in an acidic fraction of the methanolic extract, and it was the first time this compound had been isolated from a natural source [39]. Table 2. summarizes the data focused on the phytochemical characterization of plants belonging to the *Cachrys* genus.

## 6. Biological Properties of *Cachrys* Species

**Figure 4:**
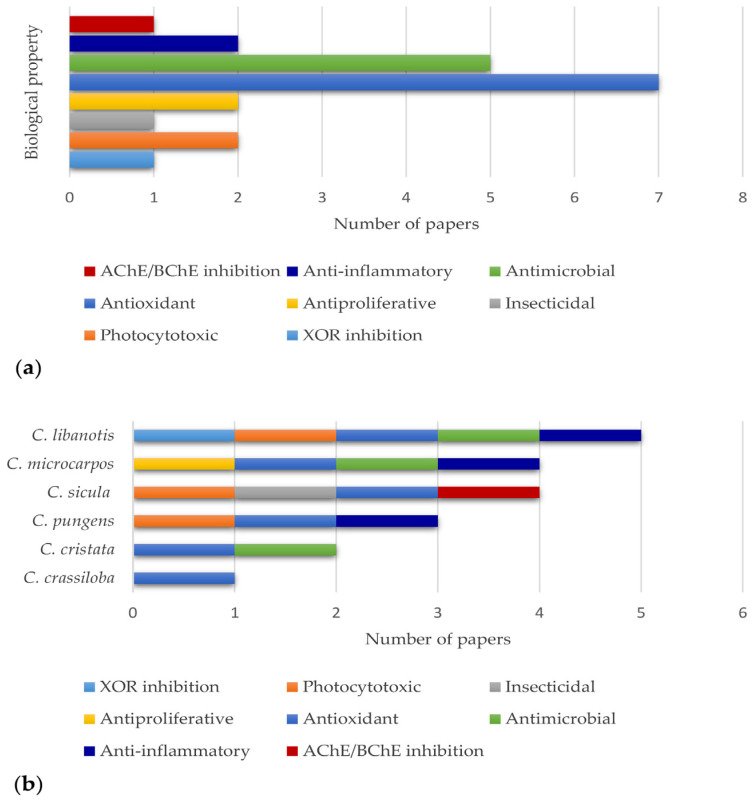
Biological properties reported for *Cachrys* spp. (**a**) Number of articles focusing on each reported biological activity. (**b**) Number of papers focusing on the biological properties of each species.

The analysis of the literature allowed us to highlight some interesting biological properties for plants belonging to the *Cachrys* genus, namely antioxidant, antibacterial, antifungal, cytotoxic, and photocytotoxic potential, together with interesting enzyme inhibitory properties and even insecticidal activity (Figure 4a). However, this genus still appears to be extremely understudied. The number of papers dealing with its biological activity is limited. We find just seven papers focusing on the antioxidant effects of *Cachrys* species and five reports about the antimicrobial potential. The number of papers dealing with the remaining activities is even lower. Overall, *C. libanotis* appears to be the most investigated species with regards to the biological potential, followed by *C. microcarpos* and *C. sicula*, for which four biological activities have been reported to date (Figure 4b).

### 6.1. Antioxidant Activity

Aouachria and coworkers explored the antioxidant potential of the hydroalcoholic root extract of *C. libanotis*, which was assessed on superoxide generated by an NADH/PMS system, DPPH test, FRAP, ferrous iron chelation, and β-carotene bleaching assays. The crude extract and its aqueous fraction showed a good chelating activity, with IC_50_ values equal to 52.6 ± 0.3 and 52.7 ± 0.2 μg/mL, respectively. The crude extract also showed the highest radical scavenging activity (IC_50_ = 0.41 ± 0.01 mg/mL). Moreover, all the fractions were effective in protecting linoleic acid from peroxidation [20].

Marrelli and colleagues investigated the in vitro potential antioxidant activity of two *Cachrys* species from southern Italy, *C. sicula* and *C. libanotis*, extracted through maceration and also using the Naviglio*^®^* extractor, an innovative solid-liquid extraction technique. The macerated extracts showed the best radical scavenging activities, with IC_50_ values of 102.13 ± 0.79 and 112.73 ± 0.88 µg/mL for *C. libanotis* and *C. sicula*, respectively, in the DPPH assay. The inhibition of lipid membrane peroxidation, investigated through β-carotene bleaching test, confirmed the trend observed for free radical scavenging activity, demonstrating that the macerated extracts significantly protected linoleic acid after 30 min of incubation (IC_50_ values equal to 16.77 ± 1.43 and 19.22 ± 1.07 µg/mL for *C. sicula* and *C. libanotis* extracts, respectively) [5].

Tahar and coworkers assessed instead the antioxidant potential of the essential oil from the leaves of *C. sicula*, using the well-established ABTS, DPPH, and metal chelating assays. A modest DPPH radical scavenging was observed, with an IC_50_ value equal to 267.94 ± 0.00 µg/mL, while the essential oil demonstrated a better antioxidant activity in the ABTS test (IC_50_ = 81.93 ± 0.00 µg/mL) [32].

Menichini and colleagues evaluated the antioxidant activity of *C. pungens* alcoholic extract and fractions. The ethyl acetate fraction showed the best radical scavenging activity, with IC_50_ equal to 12.15 ± 0.02 µg/mL. Consistently, the same fraction as well as the total extract showed the best linoleic acid protective activity in the β-carotene bleaching assay, with IC_50_ values equal to 8.33 ± 0.33 and 9.16 ± 0.27 µg/mL after 30 min, respectively. The same trend was observed after 60 min of incubation [6].

Extracts prepared starting from the aerial parts of two *C. crassiloba* (Boiss.) Meikle accessions collected in Turkey were investigated for their antioxidant potential through the DPPH assay. The extracts neutralized the DPPH radical, with percentages of inhibition equal to 67.611% and 81.281%, respectively [40].

Matejic and coworkers investigated the antioxidant potential of the aerial parts and fruits of *C. cristata* extracts by means of both the DPPH and ABTS assays. The fruit water extract showed the best radical scavenging activity (IC_50_ = 1.784 mg/mL), followed by the methanolic extracts of the fruits and aerial parts (IC_50_ values of 3.347 and 4.058 mg/mL, respectively). As regards the ABTS test, the acetone extract of *C. cristata* aerial parts showed the highest antioxidant potential, with a value of 3 mg of vitamin C per mL of extract [41].

*C. microcarpos* (synonym *Hippomarathrum microcarpum*) essential oil and methanolic extract were evaluated for their antioxidant activity by means of the DPPH and linoleic acid assays. The extract did not show any radical scavenging activity, while the essential oil exhibited an IC_50_ value of 10.690.0 ± 0.5 µg/mL (DPPH assay) [34].

### 6.2. Antimicrobial Activity

The antibacterial activity of *C. libanotis* was tested on different bacterial strains by Aouachria and colleagues. The hydroalcoholic root crude extract was effective against *Pseudomonas aeruginosa*, *Staphylococcus aureus*, and *Bacillus cereus*, while its chloroform and ethyl acetate fractions showed an antibacterial effect against *Bacillus cereus*, *Enterococcus faecalis*, and *Lysteria monocytogenes* [20].

Matejic and colleagues investigated the antimicrobial activity of *C. cristata* extracts against different strains of Gram-positive and Gram-negative bacteria. The ethyl acetate extract of *C. cristata* aerial parts showed both an inhibitory and a bactericidal activity against Gram-positive bacteria in concentrations ranging from 0.78 to 6.25 mg/mL, while inhibitory effects of the same extract were assessed against *Pseudomonas aeruginosa* without exerting any bactericidal activity at the highest tested dose [41].

*Cachrys cristata* leaf essential oil exerted antibacterial activity against *Staphylococcus aureus*, *Micrococcus luteus*, and *Staphylococcus epidermidis* bacteria. The stem essential oil produced by hydrodistillation starting from the same species was effective against *Staphylococcus aureus* Gram-positive bacteria, and showed a quite good activity against *Candida albicans* [33].

Ozer and colleagues investigated the antimicrobial and antifungal properties of *C. microcarpos* essential oil and methanolic extract. The samples exerted antimicrobial activity against eight and two bacterial strains, respectively. As the antifungal activity was also assessed, the essential oil showed the best activity by inhibiting one yeast (*Candida albicans*) and nine fungal strains [34].

Karakaya and colleagues also tested the essential oil from *C. microcarpos* aerial parts for its antimicrobial activity, reporting efficacy *against Candida albicans* and *Staphylococcus aureus* strains [35].

### 6.3. Anti-Inflammatory Potential

Perri and coworkers (2022) investigated the in vitro potential anti-inflammatory properties of two different *Cachrys* species collected in southern Italy and extracted through the Naviglio*^®^* instrument. *C. libanotis* and *C. pungens* extracts were tested for the inhibition of pro-inflammatory cytokines (TNF-α and IL-6) and NO mediator in RAW 264.7 cells activated with LPS. The data showed that both the extracts significantly inhibited the release of TNF-α and IL-6, and the production of NO. Moreover, *C. pungens* induced the release of the anti-inflammatory cytokine IL-10. Extracts were also tested for their ability to inhibit the JAK/STAT signaling pathway: both the tested samples inhibited the phosphorylation of JAK2 and STAT3 proteins, with the *C. pungens* extract being the most effective one [9].

*C. microcarpos* (synonym *Hippomarathrum microcarpum*) ethanolic extract demonstrated anti-inflammatory effects in carrageenan-induced oedema in rats, showing, at high doses, good anti-inflammatory activity and decreasing inflammation rates better than diclofenac [42].

### 6.4. Antiproliferative Activity

The antiproliferative effects of *Hippomarathrum microcarpum* (synonym *Cachrys microcarpos* M.Bieb.) aerial parts extract were tested on human prostate cancer cells (PC-3) by using the MTT assay. Both the ethanolic and the aqueous extracts inhibited cell viability after 24 h, with IC_50_ values of 12.99 and 23.34 mg/mL, respectively [43].

De Leo and colleagues tested for their antiproliferative activity three pure coumarins they isolated from the leaves of *C. sicula* L.: the new coumarin 8-hydroxymethylpsoralen and the known compounds sprengelianin and oxypeucedanin. The cytotoxic effects were assessed on Jurkat and HeLa cancer cell lines. However, 8-hydroxymethylpsoralen was the only compound demonstrating a moderate inhibitory activity [30].

### 6.5. Photocytotoxic Activity

The photocytotoxic activity observed for *Cachrys* species is linked to their furanocoumarins content [44].

Growing evidence underlines the anticancer potential of furanocoumarins. The general mechanism by which these compounds exert their effects on cancer cells is based on cell cycle blockage and programmed death, like apoptosis or autophagy [45]. Moreover, experimental studies have shown that furanocoumarins activate multiple signaling pathways, also leading to antioxidant and antimetastatic effects, and cell cycle arrest in malignant cells. Furanocoumarins have also been demonstrated to show synergistic potential with anticancer drugs [46].

Beside their antiproliferative effects, furanocoumarins are very interesting molecules since they are useful in photochemotherapy, a promising therapeutic approach in anticancer research which combines the action of a light source and a chemical photosensitizer [26,47].

PUVA therapy (Psoralen + UVA therapy) is based on the administration of psoralens followed by exposure to UVA radiation [44]. Beside its use in the treatment of dermatological diseases such as psoriasis and vitiligo, it is used in the treatment of cutaneous T-cell lymphoma [48,49,50,51].

Menichini and colleagues investigated the photocytotoxicity of *C. pungens* methanolic extract and its fractions on A375 melanoma cells irradiated at 365 nm at a dose of 1.08 J/cm^2^. The chloroform and coumarinic fractions exerted the best photocytotoxic activity, with IC_50_ values equal to 0.286 ± 0.067 and 0.209 ± 0.033 µg/mL, respectively, while they did not affect the viability of nonirradiated cells [6].

*C. sicula* and *C. libanotis* extracts photocytotoxic effects were evaluated on C32 human melanoma cells irradiated with UV light (365 nm) for 1 h at a dose of 1.08 J/cm^2^. Both the aerial parts methanolic extracts obtained throug maceration and the Naviglio*^®^* extractor were evaluated. The extracts obtained using the Naviglio*^®^* extractor significantly inhibited cell viability, with IC_50_ values of 3.16 ± 0.21 and 8.83 ± 0.20 µg/mL for *C. libanotis* and *C. sicula*, respectively. These two samples were also demonstrated to induce up-regulation of apoptotic signals such as Bcl2-associated X protein (BAX), and poly ADP-ribose polymerase (PARP) cleavage. Moreover, the apoptotic responses were also verified, and these samples proved to be more photoactive, causing a greater upregulation of the p21 protein in the presence of UVA radiation compared to the extracts obtained through traditional maceration [5].

### 6.6. Acetylcholinesterase (AChE) and Butyrylcholinesterase (BChE) Inhibitory Activity

Acetylcholinesterase (AChE) is the key enzyme in the breakdown of acetylcholine, and its inhibition is a target for the treatment of Alzheimer’s disease and other neurological disorders including senile dementia, myasthenia gravis, and ataxia [52]. Butyrylcholinesterase (BuChE) plays a minor role in regulating brain acetylcholine levels, but the activity of this enzyme increases in patients with Alzheimer*’*s disease (AD) [53]. Different plant extracts have been demonstrated to exert interesting inhibitory effects on these enzymes [52,54]. Tahar and coworkers evaluated the inhibitory properties of *C. sicula* on the acetylcholinesterase (AChE) and butyrylcholinesterase (BChE) enzymes. The essential oil was obtained from the hydrodistillation of the leaf of the plant, and the biological activity was measured using acetylthiocholine iodide and butyrylthiocholine chloride as substrates of the reaction. *C. sicula* essential oil exerted a good inhibitory activity on BChE, with an IC_50_ value equal to 91.90 ± 0.00 µg/mL, and a mild inhibitory activity on AChE (IC_50_ = 169.91 ± 0.00 µg/mL) [32].

### 6.7. Xanthine Oxidoreductase (XOR) Inhibitory Activity

The xanthine oxidoreductase (XOR) enzyme catalyzes the oxidative hydroxylation of hypoxanthine to xanthine and xanthine to uric acid and it is considered a target for the treatment of hyperuricemia and gout [55].

Aouachria and coworkers demonstrated a good xanthine oxidoreductase (XOR) inhibitory effect for *C. libanotis* root extract. To perform the experiments, the enzyme was purified from bovine milk and the inhibitory activity was measured using a spectrophotometric method, measuring uric acid production at 295 nm. Allopurinol was used as a positive control. The roots of the plants were powdered with a traditional mill and extracted with a hydroalcoholic solution. The raw extract was then fractionated using solvents with increasing polarity with liquid-liquid extraction, to achieve the corresponding hexane, chloroform, and ethyl acetate fractions. A good xanthine oxidoreductase (XOR) inhibitory effect was observed for the ethyl acetate fraction, with a reported IC_50_ value equal to 0.11 mg/mL [20].

### 6.8. Insecticidal Activity

Beside all the previous mentioned studies concerning the health beneficial properties of *Cachrys* species, an interesting study was reported by Pascual-Villalobos and Robledo, who assessed the potential anti-insect activity of a number of Mediterranean species, including *C. sicula* L. The stems were extracted with three different solvents: hexane, acetone, and methanol, and samples were tested for anti-insect activity using the insect’s growth and/or feeding inhibition. The insecticidal activity was tested on the stored grain pest *Tribolium castaneum* Herbst. *Cachrys* samples were incorporated into the diet at 0.05% concentration and larval growth inhibition and mortality were measured. A repellency bioassay was also performed. *C. sicula* hexane extract showed insecticidal activity on *Tribolium castaneum* Herbst. Moreover, a repellent index value over 50 (over a range from 0 to 100) was observed, indicating that *Tribolium castaneum* larvae were repelled compared to the control after 2 and 24 h [56].

The available data on the biological activity of *Cachrys* spp. are reported in Table 3.

## 7. Phytotoxicity and Toxicological Profile

*C. pungens* was also interestingly investigated for its potential use in sustainable agriculture, as a source of natural compounds useful for weed control. The aerial parts methanolic extract and its fractions were tested in vitro on the seed germination and root elongation of *Lectuca sativa* L. The most active samples, the chloroform and ethyl acetate fractions, were also tested for their effects on the common weeds *Lolium perenne*, *Amaranthus retroflexus*, and *Echinochloa crus-galli*. *Amaranthus retroflexus* was particularly sensitive to *C. pungens* samples, with ED_50_ values equal to 2.92 and 3.21 mg/mL for the chloroform and ethyl acetate fractions, respectively [38].

Some skin damage has been related to plants belonging to *Cachrys* genus. Ena and colleagues, already in 1989, investigated the phytochemical content of *C. libanotis* L., focusing on the presence of furanocoumarins, considered the commonest non allergic phototoxic agents. The authors described some cases of phytodermatitis following contact with the flowering plant [57]. In 1991, the same authors identified from the alcoholic extract of *C. libanotis* the furanocoumarins 5-methoxy-, 8-methoxy-, and 5,8-dimethoxypsoralen as the substances responsible for the observed phytodermatitis [58].

## 8. Other *Cachrys* Species

Different studies have focused on the phytochemical and biological properties of some *Cachrys* species which, unlike the previous classification, are currently considered members of the *Prangos* genus. The *Cachrys* group, actually, is divided into several genera, including *Cachrys*, *Prangos*, *Alocacarpum*, *Bilacunaria*, *Ferulago*, *Diplotaenia*, *Eriocycla*, and *Azilia* [59,60].

For example, in 1972, Ignat’eva and colleagues described the presence of a series of coumarins in the root extracts of *Cachrys pubescens* (Pall.) Schischnk., nowadays a synonym of *Prangos odontalgica* (Pall.) Herrnst. & Heyn [61].

Abad and coworkers described the biological potential of some furanocoumarins isolated from *Cachrys trifida* Mill [62]. Beside the name, this species is actually a synonym of *Prangos trifida* (Mill.) Herrnst. & Heyn, and it is currently considered a member of the *Prangos* group [3]. Abad and colleagues evaluated the potential role of three naturally occurring compounds in this species, namely imperatorin, isoimperatorin, and prantschimgin, in inhibiting some macrophage functions involved in the inflammatory process. The three furanocoumarins were assayed in two experimental systems: calcium ionophore A23187-stimulated mouse peritoneal macrophages, a source of COX-1 and 5-LOX enzymes, and elicited mouse peritoneal LPS-stimulated macrophages, for testing the inhibitory properties on COX-2 and iNOS. All the tested furanocoumarins were effective on 5-lipoxygenase. Furthermore, imperatorin and isoimperatorin inhibited the COX-1- and COX-2-catalyzed release of prostaglandin E_2_, and imperatorin also inhibited nitric oxide production [62].

The phytochemical content of the essential oil from the same species, *C. trifida* (*Prangos trifida*) was also assessed by Palá-Paúl and colleagues [63].

One of the most investigated species previously considered a *Cachrys* species, and at present belongs in the *Prangos* genus, is *Cachrys ferulacea* Calest., a synonym of *Prangos ferulacea* Lindl. [3]. This orophilous species of the eastern Mediterranean and western Asia has been investigated for its phytochemical content, with coumarins and flavonoids being the main compounds, and different biological properties have also been verified, as highlighted by Bruno and coworkers [59].

Bertoli and colleagues characterized the essential oils from the whole fruits, seeds, and pericarps of *Prangos ferulacea* Lindl (synonym of *Cachrys ferulacea*). α-Pinene was the main identified terpene in the fruit and pericarp essential oils, being present in percentages of 18.2% and 14.2%, respectively, while sabinene was the most abundant compound in the seed essential oil (18.7%), followed by limonene and α-pinene (18.5 and 18.4%, respectively). The coumarin osthol was only assessed in the fruit and pericarp essential oils, in percentages equal to 0.8% and 1.0%, respectively [64]. Camarda and colleagues investigated *Cachrys ferulacea* fruits collected in Sicily, southern Italy, extracted through Soxhlet in ethyl acetate. Here, osthol, bergapten, imperatorin, and isoimperatorin were isolated and identified [65].

Pistelli and colleagues evaluated *Cachrys ferulacea* seeds and roots from Sardinia, extracted through dichloromethane and hexane, respectively. Six compounds: decursin, heraclenin, imperatorin, isoimperatorin, osthol, and oxypeucedanin were isolated and identified both in the seed and root extracts [66]. The same research group investigated *Cachrys ferulacea* fruit extracts and isolated umbelliferose, a trisaccharide also found in *Angelica archangelica* L. subsp. *norvegica* (Rupr.) Nordh roots, *Astrantia major* L. fruits, and *Pimpinella saxifrage* L. [67].

Badalamenti and coworkers investigated the essential oils from the leaves and flowers of a local accession from Sicily (Italy), and the phytochemical investigation they carried out allowed them to identify a new chemotype, characterized by a large amount of (*Z*)-β-ocimene. Moreover, the essential oils were proven to exert a good antioxidant activity, inducing a decrease in ROS and an increase in the activity of superoxide dismutase (SOD), catalase (CAT), and glutathione S-transferase (GST) in opsonized zymosan (OZ)-stimulated human polymorphonuclear cells (PMNs) [68]. Other studies focused on the phytochemical content and/or the biological properties of this species. Bagherifar and coworkers, for example, analyzed the chemical composition of the essential oils from different Iranian accessions. The same authors also reported a good radical scavenging activity for these samples [69].

Moreover, *C. ferulacea*‘s potential in vitro anti-inflammatory activity was evaluated in RAW 264.7 cell lines stimulated with LPS by evaluating the presence of pro-inflammatory cytokines and mediators in the cell medium. The aerial parts extract obtained with the Naviglio^®^ extractor significantly inhibited the production of the pro-inflammatory mediator NO, and down-regulated the expression of p-JAK2 in a dose dependent-manner, making it the most effective species for anti-inflammatory activity, when compared to *C. libanotis* and *C. pungens* [9]. The same aerial part extract was also investigated for its furanocoumarins content with GC-MS analyses, with xanthotoxin, bergapten, and isopimpinellin accounting for 0.42 ± 0.05, 0.57 ± 0.04, and 0.050 ± 0.001 mg/50 mg of the extract, respectively [9]. Moreover, according to Dokovic and colleagues, 3,5-nonadiyne, a compound isolated from *Cachrys ferulacea* root essential oil, inhibited NO production in macrophages in a dose-dependent manner, with an IC_50_ value equal to 6.7 ± 0.8 µM, without showing any toxicity in rats [70].

## 9. Concluding Remarks and Future Perspectives

The taxonomy of the *Cachrys* genus is complex and the classification has been somewhat confused. For this reason, some papers reporting in their title the name “*Cachrys*” focused on plants species that, after more than one revision of the taxonomy, are currently included in different genera. According to the present classification, this review paper focused on the phytochemical properties and the demonstrated biological activity of the currently recognized *Cachrys* species, and on the whole, 34 papers were included. The analysis of the literature showed an increasing interest towards these plants, which showed, beside other phytochemical classes, a high content of terpenoids and coumarins, particularly furanocoumarins. To date, the reported phytochemical investigations allowed the identification of 177 structurally different components. The remarkable furanocoumarins content which characterizes *Cachrys* species is particularly interesting, as these compounds are acquiring particular importance in photodynamic therapy, because of their photosensitizing properties, and as a consequence, they appear to be a potential source of new drugs in different fields of application, such as dermatology and oncology. On the other side, some toxic effects, e.g., phytodermatitis, have been related to contact with *Cachrys* species due to the presence of these same compounds.

Based on the available findings, *Cachrys* appears to be a promising genus, with some potential health beneficial properties. Different biological properties have been reported to date for these species, such as antioxidant, antimicrobial, and anti-inflammatory effects, cytotoxic and photocytotoxic potential, as well as enzyme inhibitory properties. Together with these health beneficial properties, insecticidal effects have also been reported for one species, namely *C. sicula* L. However, the analysis of the literature allowed us to highlight the limited number of studies concerning the biological properties of this genus. The antioxidant and the antimicrobial effects were the most investigated properties, but just seven papers focused on the antioxidant effects of the *Cachrys* species and just five reports dealt with the antimicrobial potential. On the other side, despite some cases of phytodermatitis having been reported, the toxicological aspects are yet to be properly explored.

Moreover, some of the plants belonging to this genus have never been investigated. Such species remain to be explored both for their chemical and pharmacological properties. For all these reasons, this genus appears to be mostly understudied, and, potentially, a number of other interesting biological activities could still be discovered, as well as interesting secondary metabolites.

In conclusion, further in-depth research on *Cachrys* species is needed. Additional studies should be aimed at identifying the effective phytoconstituents of crude extracts responsible for the observed biological effects, by bioactivity guided isolation. Furthermore, toxicology and pharmacokinetic studies should also be useful in the future investigations.

Moreover, the “omics” technologies, including genomics, transcriptomics, and metabolomics, could allow even better elucidation of the biomarkers of different species, and of the relationships between metabolite biosynthesis and genetic evolution of a genus with such a difficult taxonomy. Species belonging to the same genus usually have some close chemical and biological characteristics. So, the study of the links between these properties and phylogeny could offer important information about *Cachrys* species and their potential therapeutic applications [71,72,73].

## Figures and Tables

**Table 1:** *Cachrys* L. genus: included species.

Species^1^	Scientific Name Status	Synonym
*C. aksekiensis* (A.Duran & B.Doğan) Hand	Accepted	*Bilacunaria aksekiensis* A.Duran & B.Doğan
*C. alpina* M.Bieb.	Accepted	-
*C. boissieri* (Reut. & Hausskn. ex Boiss.) Hand	Accepted	*Bilacunaria boissieri* (Reut. & Hausskn. ex Boiss.) Pimenov & V.N.Tikhom; *Hippomarathrum boissieri* Reut. & Hausskn. ex Boiss.
*C. caspica* (DC.) Menitsky	Accepted	*Bilacunaria caspia* (DC.) Pimenov & V.N.Tikhom; *Echinophora caspica* DC.; *Hippomarathrum caspicum* (DC.) Grossh.
*C. crassiloba* (Boiss.) Meikle	Accepted	*Hippomarathrum crassilobum* Boiss. & Heldr.; *Peucedanum veneris* Kotschy
*C. cristata* DC.	Accepted	*Cachrys echinophora* Guss. *Hippomarathrum cristatum* Boiss. *Hippomarathrum cristatum* var. pauciradiatum Heldr. & Halácsy *Hippomarathrum libanotis* var. cristatum (DC.) Maire *Hippomarathrum pauciradiatum* (Heldr. & Halácsy) Halácsy *Trachymarathrum siculum* Tausch
*C. korolkowi* Regel & Schmalh.	Ambiguous/ Doubtful	-
*C. libanotis* L.	Accepted	*Cachrydium libanotis* Link; *Cachrys humilis* Schousb.; *Cachrys linearia* Mill.; *Cachrys peucedanoides* Desf.; *Cachrys pterochlaena* var. leiocarpa Coss.; *Cachrys sphaerocarpa* Ten.; *Crithmum libanotis* Link; *Hippomarathrum bocconei* Boiss.; *Hippomarathrum bocconei* var. denticulatum Andr.; *Hippomarathrum libanotis* W.D.J.Koch ex DC.; *Hippomarathrum libanotis* subsp. bocconei (Boiss.) Maire; *Hippomarathrum libanotis* var. faurei Maire; *Lophocachrys echinophora* Bertol.; *Smyrnium libanotis* Crantz
*C. microcarpos* M.Bieb.	Accepted	*Bilacunaria microcarpa* (M.Bieb.) Pimenov & V.N.Tikhom; *Cachrys amplifolia* Ledeb.; *Cachrys crispa* Pers.; *Cachrys libanotis* Salzm. ex Ball; *Cachrys longiloba* DC.; *Cachrys microcarpa* M.Bieb.; *Cachrys nudicaulis* Godet ex DC.; *Hippomarathrum amplifolium* Ledeb. ex C.A.Mey.; *Hippomarathrum crispum* W.D.J.Koch; *Hippomarathrum longilobum* B.Fedtsch. ex Grossh.; *Hippomarathrum microcarpum* (M.Bieb.) Petrov
*C. nematoloba* Rech.f. & Riedl	Ambiguous/ Doubtful	-
*C. pabularia* (Lindl.) Herrnst. & Heyn	Unchecked	-
*C. pungens* Jan ex Guss	Accepted	*Hippomarathrum libanotis* var. pungens (Jan ex Guss.) Fiori; *Hippomarathrum pungens* Boiss. ex Pomel; *Hippomarathrum pungens* Lojac.
*C. scabra* (Fenzl) Meikle	Accepted	*Bilacunaria scabra* (Fenzl) Pimenov & V.N.Tikhom.; *Ferula scabra* Fenzl; *Ferulago scabra* Fenzl; *Hippomarathrum scabrum* Boiss.
*C. sibirica* Turcz. ex Ledeb.	Unchecked	-
*C. sicula* L.	Accepted	*Cachrys crispata* Pomel; *Cachrys pterochlaena* DC.; *Hippomarathrum bocconei* subsp. *crispatum* (Pomel) Batt.; *Hippomarathrum bocconei* var. brachylobum Batt.; *Hippomarathrum brachylobum* (Batt.) Sennen & Mauricio; *Hippomarathrum crispatum* Pomel; *Hippomarathrum libanotis* subsp. *pterochlaenum* (Boiss.) Maire; *Hippomarathrum libanotis* var. crispatum (Pomel) Maire; *Hippomarathrum libanotis* var. crispulum Maire; *Hippomarathrum pterochlaenum* Boiss.; *Hippomarathrum siculum* Hoffmanns. & Link; *Seseli brachylobum* (Batt.) M.Hiroe; *Smyrnium hispidum* Crantz; *Ulospermum siculum* Link

^1^ Taxonomy according to The World Flora Online (WFO) database (http://www.worldfloraonline.org/, accessed on 28 November 2022) [3].

**Table 2:** Phytochemical constituents identified in *Cachrys* spp.

Compound Name	Species	Plant Part	Extract	Analysis	Ref.
**Coumarins**					
Bergapten	*C. libanotis* L.	aerial parts	MeOH extracts (maceration and Naviglio^®^)	GC-MS ^1^	[5]
	*C. pungens* Jan ex Guss	aerial parts	MeOH extract	GC-MS, GC ^2^	[6]
	*C. sicula* L.	aerial parts	MeOH extracts (maceration and Naviglio^®^)	GC-MS	[5]
		umbels	benzene extract	NMR ^3^, IR ^4^, MS ^5^	[4]
Columbianetin	*C. pungens* Jan ex Guss	aerial parts	MeOH extract	GC-MS	[6]
Heraclenin	*C. caspica* (DC.) Menitsky	root	CHCl_3_ extract	NMR	[28]
8-Hydroxymethylpsoralen	*C. sicula* L.	leaves	EtOAc extract	NMR	[30]
Isogeijerin	*C. libanotis* L.	aerial parts	MeOH extracts (maceration and Naviglio^®^)	GC-MS	[5]
Imperatorin	*C. sicula*. L.	umbels, root	benzene extract	NMR, IR, MS	[4]
Isoimperatorin	*C. caspica* (DC.) Menitsky	root	CHCl_3_ extract	IR	[28]
	*C. sicula*. L.	umbels	benzene extract	NMR, IR, MS	[4]
Isooxypeucedanin	*C. pungens* Jan ex Guss	aerial parts	MeOH extract	GC-MS, GC	[6]
Isopimpinellin	*C. libanotis* L.	aerial parts	MeOH extracts (maceration and Naviglio^®^)	GC-MS	[5]
	*C. pungens* Jan ex Guss	aerial parts	MeOH extract	GC-MS, GC	[6]
	*C. sicula* L.	aerial parts	MeOH extracts (maceration and Naviglio^®^)	GC-MS	[5]
		umbels	benzene extract	NMR, IR, MS	[4]
Jatamansin	*C. pungens* Jan ex Guss	aerial parts	MeOH extract	GC-MS	[6]
*tert*-*O*-methylheraclenol	*C. sicula*. L.	umbels	benzene extract	NMR, IR, MS	[4]
3-Methylsuberosine	*C. pungens* Jan ex Guss	aerial parts	MeOH extract	GC-MS, GC	[10]
Osthole	*C. caspica* (DC.) Menitsky	root	CHCl_3_ extract	IR	[28]
	*C. libanotis* L.	aerial parts	MeOH extracts	GC-MS	[5]
Oxypeucedanin	*C. caspica* (DC.) Menitsky	root	CHCl_3_ extract	IR	[28]
	*C. sicula* L.	leaves	EtOAc extract	NMR	[30]
		root	benzene extract	NMR, IR, MS	[4]
Pabulenol	*C. sicula*. L.	root	benzene extract	NMR, IR, MS	[4]
Pangelin	*C. caspica* (DC.) Menitsky	root	CHCl_3_ extract	NMR	[28]
Psoralen	*C. pungens* Jan ex Guss	aerial parts	MeOH extract	GC-MS, GC	[6]
Prantschimgin	*C. sicula*. L.	umbels, root	benzene extract	NMR, IR, MS	[4]
Salaxin	*C. sicula*. L.	root	benzene extract	NMR, IR, MS	[4]
Seselin	*C. libanotis* L.	aerial parts	MeOH extract	GC-MS	[5]
Sprengelianin	*C. sicula* L.	leaf	EtOAc extract	NMR	[30]
		umbels, root	benzene extract	NMR, IR, MS	[4]
Suberosin	*C. libanotis* L.	aerial parts	MeOH extract	GC-MS	[5]
Ulopterol	*C. cristata* DC.	-	CHCl_3_ extract	IR, NMR	[29]
	*C. sicula*. L.	umbels	benzene extract	NMR, IR, MS	[4]
Umbelliferone	*C. caspica* (DC.) Menitsky	root	CHCl_3_ extract	IR	[28]
Xanthotoxin	*C. libanotis* L.	aerial parts	MeOH extracts (maceration and Naviglio*^®^*)	GC-MS	[5]
	*C. pungens* Jan ex Guss	aerial parts	MeOH extract	GC-MS, GC	[6]
	*C. sicula* L.	aerial parts	MeOH extracts (maceration and Naviglio*^®^*)	GC-MS	[5]
		umbels	Benzene extract	NMR, IR, MS	[4]
2-Methyl-2-butenoic acid 9,10-dihydro-8,8-dimethyl-2-oxo-2H,8Hbenzo[1,2- b:3,4-b0]dipyran-9-yl ester	*C. libanotis* L.	aerial parts	MeOH extract (Naviglio*^®^*)	GC-MS	[5]
	*C. sicula* L.	aerial parts	MeOH extract (Naviglio*^®^*)	GC-MS	[5]
**Terpenoids**					
Anethole	*C. cristata* DC.	fruit	EO ^6^	GC-MS	[23]
	*C. sicula* L.	aerial parts	MeOH extract (Naviglio^®^)	GC-MS	[5]
Aromadendrene	*C. cristata* DC.	leaf	EO	GC-MS	[33]
	*C. sicula* L.	aerial parts	EO	GC-MS	[15]
Bicycloelemene	*C. libanotis* L.	aerial parts	EO	GC-MS	[7]
bicyclogermacrene	*C. alpina* M.Bieb.	fruit	EO	GC-MS	[21]
	*C. boissieri* (Reut. & Hausskn. ex Boiss.) Hand	aerial parts	EO	GC-MS	[37]
	*C. cristata* DC.	leaf, stem, fruit	EO	GC-MS	[23,33]
		whole plant	EO	GC-MS	[31]
	*C. sicula* L.	leaf	EO	GC-MS	[32]
β-bisabolene	*C. cristata* DC.	leaf, root, fruit	EO	GC-MS	[23,33]
	*C. microcarpos* M.Bieb.	flower, leaf, aerial parts	EO	GC-MS	[8,36]
	*C. pungens* Jan ex Guss	aerial parts	MeOH extract	GC-MS	[6]
γ-bisabolene	*C. microcarpos* M.Bieb.	leaf	EO	GC-MS	[34]
α-bisabolol	*C. cristata* DC.	leaf, root, fruit	EO	GC-MS	[23,33]
epi-α-bisabolol	*C. sicula* L.	leaf	EO	GC-MS	[32]
borneol	*C. boissieri* (Reut. & Hausskn. ex Boiss.) Hand	aerial parts	EO	GC-MS	[37]
	*C. microcarpos* M.Bieb.	leaf	EO	GC-MS	[34]
		aerial parts	EO	GC-MS	[8]
	*C. sicula* L.	aerial parts	EO	GC-MS	[15]
bornyl acetate	*C. alpina* M.Bieb.	fruit	EO	GC-MS	[21]
	*C. boissieri* (Reut. & Hausskn. ex Boiss.) Hand	aerial parts	EO	GC-MS	[37]
	*C. cristata* DC.	leaf, stem, root, fruit	EO	GC-MS	[23,33]
	*C. microcarpos* M.Bieb.	flower, leaf, aerial parts	EO	GC-MS	[8,34,36]
	*C. sicula* L.	aerial parts, leaf	EO	GC-MS	[15,32]
α-bourbonene	*C. boissieri* (Reut. & Hausskn. ex Boiss.) Hand	aerial parts	EO	GC-MS	[37]
	*C. cristata* DC.	fruit	EO	GC-MS	[23]
β-bourbonene	*C. alpina* M.Bieb.	fruit	EO	GC-MS	[21]
	*C. boissieri* (Reut. & Hausskn. ex Boiss.) Hand	aerial parts	EO	GC-MS	[37]
	*C. cristata* DC.	fruit	EO	GC-MS	[23]
	*C. microcarpos* M.Bieb.	flower, leaf, aerial parts	EO	GC-MS	[8,34,36]
	*C. sicula* L.	aerial parts	EO	GC-MS	[15]
γ-cadinene	*C. alpina* M.Bieb.	fruit	EO	GC-MS	[21]
	*C. boissieri* (Reut. & Hausskn. ex Boiss.) Hand	aerial parts	EO	GC-MS	[37]
	*C. cristata* DC.	leaf, fruit	EO	GC-MS	[23,33]
	*C. microcarpos* M.Bieb.	leaf	EO	GC-MS	[34]
	*C. pungens* Jan ex Guss	aerial parts	MeOH extract	GC-MS	[6]
δ-cadinene	*C. alpina* M.Bieb.	fruit	EO	GC-MS	[21]
	*C. boissieri* (Reut. & Hausskn. ex Boiss.) Hand	aerial parts	EO	GC-MS	[37]
	*C. cristata* DC.	leaf, stem, fruit	EO	GC-MS	[23,33]
		whole plant	EO	GC-MS	[31]
	*C. libanotis* L.	aerial parts	EO	GC-MS	[7]
	*C. microcarpos* M.Bieb.	flower, leaf, aerial parts	EO	GC-MS	[34,35,36]
	*C. sicula* L.	aerial parts, leaf	EO	GC-MS	[15,32]
α-cadinol	*C. alpina* M.Bieb	fruit	EO	GC-MS	[21]
	*C. boissieri* (Reut. & Hausskn. ex Boiss.) Hand	aerial parts	EO	GC-MS	[37]
	*C. cristata* DC.	fruit	EO	GC-MS	[23]
	*C. microcarpos* M.Bieb.	aerial parts	EO	GC-MS	[8]
	*C. sicula* L.	aerial parts, leaf	EO	GC-MS	[15,32]
Τ-cadinol	*C. alpina* M.Bieb	fruit	EO	GC-MS	[21]
	*C. boissieri* (Reut. & Hausskn. ex Boiss.) Hand	aerial parts	EO	GC-MS	[37]
	*C. sicula* L.	leaf	EO	GC-MS	[32]
α-calacorene	*C. boissieri* (Reut. & Hausskn. ex Boiss.) Hand	aerial parts	EO	GC-MS	[37]
	*C. libanotis* L.	aerial parts	EO	GC-MS	[7]
β-calacorene	*C. microcarpos* M.Bieb.	leaf	EO	GC-MS	[34]
*cis*-calamenene	*C. libanotis* L.	aerial parts	EO	GC-MS	[7]
calarene	*C. libanotis* L.	aerial parts	EO	GC-MS	[7]
camphene	*C. boissieri* (Reut. & Hausskn. ex Boiss.) Hand	aerial parts	EO	GC-MS	[37]
	*C. cristata* DC.	leaf, stem, root, fruit	EO	GC-MS	[23,33]
	*C. microcarpos* M.Bieb.	aerial parts, leaf, flower	EO	GC-MS	[8,35,36]
	*C. sicula* L.	aerial parts, leaf	EO	GC-MS	[15,32]
camphor	*C. microcarpos* M.Bieb.	leaf	EO	GC-MS	[34]
δ-3-carene	*C. cristata* DC.	leaf, stem, root	EO	GC-MS	[33]
	*C. boissieri* (Reut. & Hausskn. ex Boiss.) Hand	aerial parts	EO	GC-MS	[37]
	*C. sicula* L.	aerial parts	EO	GC-MS	[15]
carvacrol	*C. alpina* M.Bieb.	fruit	EO	GC-MS	[21]
	*C. cristata* DC.	fruit	EO	GC-MS	[23]
	*C. boissieri* (Reut. & Hausskn. ex Boiss.) Hand	aerial parts	EO	GC-MS	[37]
	*C. libanotis* L.	aerial parts	EO	GC-MS	[7]
	*C. microcarpos* M.Bieb.	flower, leaf	EO	GC-MS	[36]
	*C. pungens* Jan ex Guss	aerial parts	MeOH extract	GC-MS	[6]
	*C. sicula* L.	aerial parts	EO	GC-MS	[15]
carvacrol methyl ether	*C. cristata* DC.	leaf, stem	EO	GC-MS	[33]
*trans*-carveol	*C. boissieri* (Reut. & Hausskn. ex Boiss.) Hand	aerial parts	EO	GC-MS	[37]
	*C. microcarpos* M.Bieb.	leaf	EO	GC-MS	[34]
carvenone	*C. cristata* DC.	leaf	EO	GC-MS	[33]
carvone	*C. microcarpos* M.Bieb.	leaf	EO	GC-MS	[34]
β-caryophyllene	*C. alpina* M.Bieb	fruit	EO	GC-MS	[21]
	*C. boissieri* (Reut. & Hausskn. ex Boiss.) Hand	aerial parts	EO	GC-MS	[37]
	*C. cristata* DC.	leaf, stem, fruit	EO	GC-MS	[23,33]
		whole plant	EO	GC-MS	[31]
	*C. microcarpos* M.Bieb.	flower, leaf, aerial parts	EO	GC-MS	[8,34,35,36]
	*C. pungens* Jan ex Guss	aerial parts	MeOH extract	GC-MS	[6]
	*C. sicula* L.	leaf	EO	GC-MS	[32]
caryophyllene oxide	*C. boissieri* (Reut. & Hausskn. ex Boiss.) Hand	aerial parts	EO	GC-MS	[37]
	*C. cristata* DC.	leaf, stem, fruit	EO	GC-MS	[23,24,25,26,27,28,29,30,31,32,33]
	*C. libanotis* L.	aerial parts	EO	GC-MS	[7]
	*C. microcarpos* M.Bieb.	flower, leaf, aerial parts	EO	GC-MS	[8,34,35,36]
	*C. sicula* L.	leaf	EO	GC-MS	[32]
1,8-cineole	*C. microcarpos* M.Bieb.	leaf	EO	GC-MS	[34]
clovene	*C. pungens* Jan ex Guss	aerial parts	MeOH extract	GC-MS	[6]
α-copaene	*C. boissieri* (Reut. & Hausskn. ex Boiss.) Hand	aerial parts	EO	GC-MS	[37]
	*C. cristata* DC.	leaf, stem, fruit	EO	GC-MS	[23,24,25,26,27,28,29,30,31,32,33]
	*C. libanotis* L.	aerial parts	EO	GC-MS	[7]
	*C. microcarpos* M.Bieb.	flower, leaf, aerial parts	EO	GC-MS	[34,35,36]
	*C. pungens* Jan ex Guss	aerial parts	MeOH extract	GC-MS	[6]
	*C. sicula* L.	leaf	EO	GC-MS	[32]
β-copaene	*C. cristata* DC.	fruit	EO	GC-MS	[23]
cryptone	*C. cristata* DC.	root	EO	GC-MS	[33]
*p*-cymene	*C. alpina* M.Bieb	fruit	EO	GC-MS	[21]
	*C. boissieri* (Reut. & Hausskn. ex Boiss.) Hand	aerial parts	EO	GC-MS	[37]
	*C. cristata* DC.	leaf, stem, root, fruit	EO	GC-MS	[23,33]
	*C. microcarpos* M.Bieb.	flower, leaf, aerial parts	EO	GC-MS	[8,34,35,36]
	*C. libanotis* L.	aerial parts	EO	GC-MS	[7]
	*C. sicula* L.	aerial parts, leaf	EO	GC-MS	[15,32]
*p*-cymenene	*C. cristata* DC.	root	EO	GC-MS	[33]
*p*-cymen-8-ol	*C. boissieri* (Reut. & Hausskn. ex Boiss.) Hand	aerial parts	EO	GC-MS	[37]
	*C. cristata* DC.	leaf, stem	EO	GC-MS	[33]
	*C. libanotis* L.	aerial parts	EO	GC-MS	[7]
	*C. microcarpos* M.Bieb.	leaf	EO	GC-MS	[34]
	*C. sicula* L.	aerial parts	EO	GC-MS	[15]
α-cubebene	*C. libanotis* L.	aerial parts	EO	GC-MS	[7]
	*C. microcarpos* M.Bieb.	flower	EO	GC-MS	[36]
	*C. pungens* Jan ex Guss	aerial parts	MeOH extract	GC-MS	[6]
	*C. sicula* L.	aerial parts	EO	GC-MS	[15]
β-cubebene	*C. boissieri* (Reut. & Hausskn. ex Boiss.) Hand	aerial parts	EO	GC-MS	[37]
	*C. cristata* DC.	leaf, stem	EO	GC-MS	[33]
	*C. libanotis* L.	aerial parts	EO	GC-MS	[7]
	*C. microcarpos* M.Bieb.	flower, leaf, aerial parts	EO	GC-MS	[8,36]
	*C. sicula* L.	leaf	EO	GC-MS	[32]
epi-cubebol	*C. boissieri* (Reut. & Hausskn. ex Boiss.) Hand	aerial parts	EO	GC-MS	[37]
	*C. sicula* L.	aerial parts	EO	GC-MS	[15]
cubebol	*C. boissieri* (Reut. & Hausskn. ex Boiss.) Hand	aerial parts	EO	GC-MS	[37]
	*C. cristata* DC.	fruit	EO	GC-MS	[23]
α-cuprenene	*C. cristata* DC.	leaf	EO	GC-MS	[33]
daucene	*C. cristata* DC.	leaf, stem	EO	GC-MS	[33]
β-elemene	*C. alpina* M.Bieb.	fruit	EO	GC-MS	[21]
	*C. cristata* DC.	leaf, stem, fruit	EO	GC-MS	[23,24,25,26,27,28,29,30,31,32,33]
	*C. microcarpos* M.Bieb.	leaf	EO	GC-MS	[34]
γ-elemene	*C. cristata* DC.	leaf, stem	EO	GC-MS	[33]
	*C. sicula* L.	aerial parts	EO	GC-MS	[15]
δ-elemene	*C. cristata* DC.	leaf	EO	GC-MS	[33]
elemol	*C. cristata* DC.	root	EO	GC-MS	[33]
eremophilene	*C. boissieri* (Reut. & Hausskn. ex Boiss.) Hand	aerial parts	EO	GC-MS	[37]
estragole	*C. sicula* L.	aerial parts	MeOH extracts (maceration and Naviglio^®^)	GC-MS	[5]
	*C. libanotis* L.	aerial parts	MeOH extracts (maceration and Naviglio*^®^*)	GC-MS	[5]
7-epi-dehydrosesquicineole	*C. cristata* DC.	leaf, stem	EO	GC-MS	[33]
4-epi-cis-dihydro-agarofuran	*C. cristata* DC.	root	EO	GC-MS	[33]
2,5-dimethoxy-*p*-cymene	*C. cristata* DC.	root	EO	GC-MS	[33]
α-farnesene	*C. boissieri* (Reut. & Hausskn. ex Boiss.) Hand	aerial parts	EO	GC-MS	[37]
	*C. cristata* DC.	leaf	EO	GC-MS	[33]
β-farnesene	*C. cristata* DC.	fruit	EO	GC-MS	[23]
	*C. pungens* Jan ex Guss	aerial parts	MeOH extract	GC-MS	[6]
	*C. sicula* L.	aerial parts	EO	GC-MS	[15]
fenchyl alcohol	*C. microcarpos* M.Bieb.	aerial parts	EO	GC-MS	[35]
	*C. cristata* DC.	leaf, stem, root	EO	GC-MS	[33]
fenchone	*C. sicula* L.	aerial parts	MeOH extract (Naviglio^®^)	GC-MS	[5]
β-funebrene	*C. cristata* DC.	fruit	EO	GC-MS	[23]
geranyl acetone	*C. cristata* DC.	fruit	EO	GC-MS	[23]
geraniol	*C. microcarpos* M.Bieb.	leaf	EO	GC-MS	[36]
germacrene B	*C. alpina* M.Bieb.	fruit	EO	GC-MS	[21]
	*C. boissieri* (Reut. & Hausskn. ex Boiss.) Hand	aerial parts	EO	GC-MS	[37]
	*C. cristata* DC.	leaf, fruit	EO	GC-MS	[23,24,25,26,27,28,29,30,31,32,33]
	*C. microcarpos* M.Bieb.	aerial parts	EO	GC-MS	[8]
	*C. sicula* L.	aerial parts	EO	GC-MS	[15]
germacrene D	*C. alpina* M.Bieb.	fruit	EO	GC-MS	[21]
	*C. boissieri* (Reut. & Hausskn. ex Boiss.) Hand	aerial parts	EO	GC-MS	[37]
	*C. cristata* DC.	leaf, stem, fruit	EO	GC-MS	[23,24,25,26,27,28,29,30,31,32,33]
		whole plant	EO	GC-MS	[31]
	*C. libanotis* L.	aerial parts	EO	GC-MS	[7]
	*C. microcarpos* M.Bieb.	flower, leaf, aerial parts	EO	GC-MS	[8,34,35,36]
	*C. sicula* L.	aerial parts, leaf	EO	GC-MS	[15,32]
β-gurjunene	*C. sicula* L.	aerial parts	EO	GC-MS	[15]
	*C. microcarpos* M.Bieb.	flower, leaf	EO	GC-MS	[36]
γ-gurjunene	*C. boissieri* (Reut. & Hausskn. ex Boiss.) Hand	aerial parts	EO	GC-MS	[37]
α-humulene	*C. alpina* M.Bieb.	fruit	EO	GC-MS	[21]
	*C. boissieri* (Reut. & Hausskn. ex Boiss.) Hand	aerial parts	EO	GC-MS	[37]
	*C. cristata* DC.	leaf, stem, fruit	EO	GC-MS	[23,24,25,26,27,28,29,30,31,32,33]
	*C. microcarpos* M.Bieb.	flower, leaf, aerial parts	EO	GC-MS	[8,34,35,36]
	*C. sicula* L.	aerial parts, leaf	EO	GC-MS	[15,32]
humulene epoxide	*C. boissieri* (Reut. & Hausskn. ex Boiss.) Hand	aerial parts	EO	GC-MS	[37]
	*C. cristata* DC.	fruit	EO	GC-MS	[23]
	*C. microcarpos* M.Bieb.	aerial parts	EO	GC-MS	[35]
kessane	*C. cristata* DC.	root, fruit	EO	GC-MS	[23,24,25,26,27,28,29,30,31,32,33]
limonene	*C. alpina* M.Bieb.	fruit	EO	GC-MS	[21]
	*C. boissieri* (Reut. & Hausskn. ex Boiss.) Hand	aerial parts	EO	GC-MS	[37]
	*C. cristata* DC.	fruit	EO	GC-MS	[23]
	*C. libanotis* L.	aerial parts	EO	GC-MS	[7]
	*C. microcarpos* M.Bieb.	Leaf, aerial parts	EO	GC-MS	[34,35]
	*C. sicula* L.	aerial parts	MeOH extracts (maceration and Naviglio^®^)	GC-MS	[5]
		aerial parts, leaf	EO	GC-MS	[15,32]
linalool	*C. microcarpos* M.Bieb.	flower, leaf	EO	GC-MS	[34,36]
	*C. sicula* L.	aerial parts, leaf	EO	GC-MS	[15,32]
α-longipinene	*C. pungens* Jan ex Guss	aerial parts	MeOH extract	GC-MS	[6]
1,3,8-*p*-menthatriene	*C. cristata* DC.	leaf, root	EO	GC-MS	[33]
	*C. sicula* L.	aerial parts	EO	GC-MS	[15]
α-muurolene	*C. alpina* M.Bieb.	fruit	EO	GC-MS	[21]
	*C. cristata* DC.	root	EO	GC-MS	[33]
γ-muurolene	*C. alpina* M.Bieb.	fruit	EO	GC-MS	[21]
	*C. boissieri* (Reut. & Hausskn. ex Boiss.) Hand	aerial parts	EO	GC-MS	[37]
	*C. cristata* DC.	stem, fruit	EO	GC-MS	[23,24,25,26,27,28,29,30,31,32,33]
	*C. microcarpos* M.Bieb.	leaf	EO	GC-MS	[36]
α-muurolol	*C. boissieri* (Reut. & Hausskn. ex Boiss.) Hand	aerial parts	EO	GC-MS	[37]
T-muurolol	*C. alpina* M.Bieb.	fruit	EO	GC-MS	[21]
	*C. boissieri* (Reut. & Hausskn. ex Boiss.) Hand	aerial parts	EO	GC-MS	[37]
β-myrcene	*C. cristata* DC.	leaf, stem, root, fruit	EO	GC-MS	[23,24,25,26,27,28,29,30,31,32,33]
		whole plant	EO	GC-MS	[31]
	*C. libanotis* L.	aerial parts	EO	GC-MS	[7]
	*C. microcarpos* M.Bieb.	aerial parts	EO	GC-MS	[8]
		leaf, flower	EO	GC-MS	[34,36]
	*C. pungens* Jan ex Guss	aerial parts	MeOH extract	GC-MS	[6]
	*C. sicula* L.	aerial parts, leaf	EO	GC-MS	[15,32]
myrtenol	*C. libanotis* L.	aerial parts	EO	GC-MS	[7]
myrtenyl acetate	*C. microcarpos* M.Bieb.	leaf, flower	EO	GC-MS	[36]
neophytadiene	*C. sicula* L.	aerial parts	MeOH extract	GC-MS	[5]
	*C. libanotis* L.	aerial parts	EO	GC-MS	[7]
	*C. pungens* Jan ex Guss	aerial parts	MeOH extract	GC-MS	[6]
β-ocimene	*C. cristata* DC.	leaf, stem, root, fruit	EO	GC-MS	[23,24,25,26,27,28,29,30,31,32,33]
	*C. microcarpos* M.Bieb.	leaf, aerial parts	EO	GC-MS	[8,34,35]
	*C. pungens* Jan ex Guss	aerial parts	MeOH extract	GC-MS	[6]
	*C. sicula* L.	aerial parts, leaf	EO	GC-MS	[15,32]
*allo*-ocimene	*C. cristata* DC.	leaf, stem	EO	GC-MS	[33]
	*C. libanotis* L.	aerial parts	EO	GC-MS	[7]
epoxy-ocimene	*C. cristata* DC.	leaf, stem	EO	GC-MS	[33]
α-phellandrene	*C. alpina* M.Bieb.	fruit	EO	GC-MS	[21]
	*C. boissieri* (Reut. & Hausskn. ex Boiss.) Hand	aerial parts	EO	GC-MS	[37]
	*C. cristata* DC.	leaf, stem, root	EO	GC-MS	[33]
	*C. microcarpos* M.Bieb.	aerial parts	EO	GC-MS	[8,9,10,11,12,13,14,15,16,17,18,19,20,21,22,23,24,25,26,27,28,29,30,31,32,33,34,35]
	*C. pungens* Jan ex Guss	aerial parts	MeOH extract	GC-MS	[6]
	*C. sicula* L.	aerial parts	EO	GC-MS	[15]
β-phellandrene	*C. alpina* M.Bieb.	fruit	EO	GC-MS	[21]
	*C. boissieri* (Reut. & Hausskn. ex Boiss.) Hand	aerial parts	EO	GC-MS	[37]
	*C. cristata* DC.	whole plant, fruit	EO	GC-MS	[23,24,25,26,27,28,29,30,31]
	*C. pungens* Jan ex Guss	aerial parts	MeOH extract	GC-MS	[6]
	*C. microcarpos* M.Bieb.	flower, leaf, aerial parts	EO	GC-MS	[8,34,35,36]
phytol	*C. cristata* DC.	whole plant	EO	GC-MS	[31]
	*C. microcarpos* M.Bieb.	aerial parts	EO	GC-MS	[8]
α-pinene	*C. alpina* M.Bieb.	fruit	EO	GC-MS	[21]
	*C. boissieri* (Reut. & Hausskn. ex Boiss.) Hand	aerial parts	EO	GC-MS	[37]
	*C. cristata* DC.	leaf, stem, root, fruit	EO	GC-MS	[23,24,25,26,27,28,29,30,31,32,33]
	*C. microcarpos* M.Bieb.	leaf, flower, aerial parts	EO	GC-MS	[8,34,35,36]
	*C. libanotis* L.	aerial parts	EO	GC-MS	[7]
	*C. sicula* L.	aerial parts, leaf	EO	GC-MS	[15,32]
β-pinene	*C. alpina* M.Bieb.	fruit	EO	GC-MS	[21]
	*C. boissieri* (Reut. & Hausskn. ex Boiss.) Hand	aerial parts	EO	GC-MS	[37]
	*C. cristata* DC.	whole plant, fruit	EO	GC-MS	[23,24,25,26,27,28,29,30,31]
	*C. libanotis* L.	aerial parts	EO	GC-MS	[7]
	*C. microcarpos* M.Bieb.	flower, leaf, aerial parts	EO	GC-MS	[8,35,36]
	*C. sicula* L.	aerial parts, leaf	EO	GC-MS	[15,32]
pinocamphone	*C. microcarpos* M.Bieb.	leaf	EO	GC-MS	[34]
pinocarvone	*C. libanotis* L.	aerial parts	EO	GC-MS	[7]
	*C. microcarpos* M.Bieb.	leaf	EO	GC-MS	[34]
piperitenone	*C. microcarpos* M.Bieb.	leaf	EO	GC-MS	[34]
piperitenone oxide	*C. microcarpos* M.Bieb.	leaf	EO	GC-MS	[34]
piperitone	*C. microcarpos* M.Bieb.	leaf	EO	GC-MS	[34]
pregeijerene	*C. cristata* DC.	fruit	EO	GC-MS	[23]
pulegone	*C. microcarpos* M.Bieb.	leaf	EO	GC-MS	[34]
sabinene	*C. alpina* M.Bieb.	fruit	EO	GC-MS	[21]
	*C. boissieri* (Reut. & Hausskn. ex Boiss.) Hand	aerial parts	EO	GC-MS	[37]
	*C. cristata* DC.	leaf, stem, root, fruit	EO	GC-MS	[23,24,25,26,27,28,29,30,31,32,33]
	*C. libanotis* L.	aerial parts	EO	GC-MS	[7]
	*C. microcarpos* M.Bieb.	flower, leaf, aerial parts	EO	GC-MS	[8,35,36]
	*C. sicula* L.	aerial parts, leaf	EO	GC-MS	[15,32]
sabinyl acetate	*C. cristata* DC.	root	EO	GC-MS	[33]
	*C. microcarpos* M.Bieb.	leaf	EO	GC-MS	[34]
	*C. sicula* L.	aerial parts	EO	GC-MS	[15]
safranal	*C. microcarpos* M.Bieb.	leaf	EO	GC-MS	[34]
selina 3,7(11)-diene	*C. cristata* DC.	leaf, stem	EO	GC-MS	[33]
α-selinene	*C. cristata* DC.	root, fruit	EO	GC-MS	[23,24,25,26,27,28,29,30,31,32,33]
β-selinene	*C. cristata* DC.	leaf, stem	EO	GC-MS	[33]
	*C. pungens* Jan ex Guss	aerial parts	MeOH extract	GC-MS	[6]
β-sesquiphellandrene	*C. cristata* DC.	leaf	EO	GC-MS	[33]
	*C. microcarpos* M.Bieb.	leaf, aerial parts	EO	GC-MS	[8,34,36]
spathulenol	*C. alpina* M.Bieb.	fruit	EO	GC-MS	[21]
	*C. boissieri* (Reut. & Hausskn. ex Boiss.) Hand	aerial parts	EO	GC-MS	[37]
	*C. cristata* DC.	leaf, stem, fruit	EO	GC-MS	[23,24,25,26,27,28,29,30,31,32,33]
	*C. libanotis* L.	aerial parts	EO	GC-MS	[7]
	*C. microcarpos* M.Bieb.	flower, leaf	EO	GC-MS	[34,36]
	*C. sicula* L.	aerial parts	EO	GC-MS	[15]
α-terpinene	*C. boissieri* (Reut. & Hausskn. ex Boiss.) Hand	aerial parts	EO	GC-MS	[37]
	*C. libanotis* L.	aerial parts	EO	GC-MS	[7]
	*C. microcarpos* M.Bieb.	aerial parts	EO	GC-MS	[8]
	*C. sicula* L.	aerial parts, leaf	EO	GC-MS	[15,32]
γ-terpinene	*C. boissieri* (Reut. & Hausskn. ex Boiss.) Hand	aerial parts	EO	GC-MS	[37]
	*C. cristata* DC.	leaf, stem, root, fruit	EO	GC-MS	[23,24,25,26,27,28,29,30,31,32,33]
		whole plant	EO	GC-MS	[31]
	*C. libanotis* L.	aerial parts	EO	GC-MS	[7]
	*C. microcarpos* M.Bieb.	aerial parts, leaf	EO	GC-MS	[8,35,36]
	*C. sicula* L.	aerial parts, leaf	EO	GC-MS	[15,32]
terpinen-4-ol	*C. cristata* DC.	leaf, stem	EO	GC-MS	[33]
	*C. libanotis* L.	aerial parts	EO	GC-MS	[7]
	*C. microcarpos* M.Bieb.	aerial parts	EO	GC-MS	[8]
	*C. sicula* L.	aerial parts	EO	GC-MS	[15]
α-terpineol	*C. microcarpos* M.Bieb.	leaf	EO	GC-MS	[34]
	*C. sicula* L.	aerial parts, leaf	EO	GC-MS	[15,32]
terpinolene	*C. alpina* M.Bieb.	fruit	EO	GC-MS	[21]
	*C. boissieri* (Reut. & Hausskn. ex Boiss.) Hand	aerial parts	EO	GC-MS	[37]
	*C. cristata* DC.	leaf, stem, root	EO	GC-MS	[33]
	*C. libanotis* L.	aerial parts	EO	GC-MS	[7]
	*C. microcarpos* M.Bieb.	aerial parts	EO	GC-MS	[8,35]
	*C. sicula* L.	aerial parts, leaf	EO	GC-MS	[15,32]
thymol	*C. boissieri* (Reut. & Hausskn. ex Boiss.) Hand	aerial parts	EO	GC-MS	[37]
	*C. libanotis* L.	aerial parts	EO	GC-MS	[7]
	*C. microcarpos* M.Bieb.	flower, leaf	EO	GC-MS	[34,36]
	*C. sicula* L.	aerial parts	EO	GC-MS	[15]
thymol methyl ether	*C. cristata* DC.	leaf, stem	EO	GC-MS	[33]
	*C. libanotis* L.	aerial parts	EO	GC-MS	[7]
2,4(10)-thujadiene	*C. microcarpos* M.Bieb.	leaf	EO	GC-MS	[34]
α-thujene	*C. boissieri* (Reut. & Hausskn. ex Boiss.) Hand	aerial parts	EO	GC-MS	[37]
	*C. cristata* DC.	leaf, stem, root	EO	GC-MS	[33]
	*C. microcarpos* M.Bieb.	aerial parts	EO	GC-MS	[8]
	*C. sicula* L.	aerial parts, leaf	EO	GC-MS	[15,32]
valencene	*C. microcarpos* M.Bieb.	leaf	EO	GC-MS	[36]
verbenene	*C. libanotis* L.	aerial parts	EO	GC-MS	[7]
verbenol	*C. boissieri* (Reut. & Hausskn. ex Boiss.) Hand	aerial parts	EO	GC-MS	[37]
	*C. microcarpos* M.Bieb.	leaf	EO	GC-MS	[34]
verbenone	*C. boissieri* (Reut. & Hausskn. ex Boiss.) Hand	aerial parts	EO	GC-MS	[37]
	*C. microcarpos* M.Bieb.	leaf	EO	GC-MS	[34]
β-vetivone	*C. cristata* DC.	leaf, stem	EO	GC-MS	[33]
zingiberene	*C. cristata* DC.	fruit	EO	GC-MS	[23]
	*C. microcarpos* M.Bieb.	flower, leaf, aerial parts	EO	GC-MS	[8,36]
zonarene	*C. cristata* DC.	leaf, stem	EO	GC-MS	[33]
**Phenylpropanoids**					
eugenol	*C. boissieri* (Reut. & Hausskn. ex Boiss.) Hand	aerial parts	EO	GC-MS	[37]
methyl eugenol	*C. cristata* DC.	fruit	EO	GC-MS	[23]
myristicin	*C. cristata* DC.	fruit	EO	GC-MS	[23]
**Phenolic compounds**					
caffeic acid	*C. pungens* Jan ex Guss	aerial parts	MeOH extract	HPTLC	[38]
catechin	*C. pungens* Jan ex Guss	aerial parts	MeOH extract	HPTLC	[38]
ferulic acid	*C. pungens* Jan ex Guss	aerial parts	MeOH extract	HPTLC	[38]
gallic acid	*C. pungens* Jan ex Guss	aerial parts	MeOH extract	HPTLC	[38]
naringin	*C. pungens* Jan ex Guss	aerial parts	MeOH extract	HPTLC	[38]
quercitrin	*C. pungens* Jan ex Guss	aerial parts	MeOH extract	HPTLC	[38]
**Fatty acids**					
behenic acid methyl ester	*C. pungens* Jan ex Guss	aerial parts	MeOH extract	GC-MS	[6]
ethyl linoleate	*C. cristata* DC.	fruit	EO	GC-MS	[23]
ethyl palmitate	*C. cristata* DC.	fruit	EO	GC-MS	[23]
ethyl oleate	*C. cristata* DC.	fruit	EO	GC-MS	[23]
lauric acid	*C. pungens* Jan ex Guss	aerial parts	MeOH extract	GC-MS	[6]
linoleic acid	*C. cristata* DC.	seed	light petroleum extract	GC-MS	[13]
	*C. pungens* Jan ex Guss	aerial parts	MeOH extract	GC-MS	[6]
α-linolenic acid	*C. libanotis* L.	aerial parts	MeOH extract	GC-MS	[5]
margaric acid	*C. pungens* Jan ex Guss	aerial parts	MeOH extract	GC-MS	[6]
methyl linoleate	*C. cristata* DC.	fruit	EO	GC-MS	[23]
methyl oleate	*C. cristata* DC.	fruit	EO	GC-MS	[23]
methyl palmitate	*C. cristata* DC.	fruit	EO	GC-MS	[23]
myristic acid	*C. cristata* DC.	fruit	EO	GC-MS	[23]
	*C. libanotis* L.	aerial parts	MeOH extract (Naviglio*^®^*)	GC-MS	[5]
myristic acid methyl ester	*C. pungens* Jan ex Guss	aerial parts	MeOH extract	GC-MS	[6]
8,11-octadecadienoic acid, methyl ester	*C. pungens* Jan ex Guss	aerial parts	MeOH extract	GC-MS	[6]
palmitic acid	*C. cristata* DC.	fruit	EO	GC-MS	[23]
	*C. libanotis* L.	aerial parts	MeOH extracts (maceration and Naviglio*^®^*)	GC-MS	[5]
	*C. microcarpos* M.Bieb.	aerial parts	EO	GC-MS	[8]
	*C. pungens* Jan ex Guss	aerial parts	MeOH extract	GC-MS	[6]
	*C. sicula* L.	aerial parts	MeOH extracts (maceration and Naviglio*^®^*)	GC-MS	[5]
pentadecanoic acid methyl ester	*C. pungens* Jan ex Guss	aerial parts	MeOH extract	GC-MS	[6]
petroselinic acid	*C. cristata* DC.	seed	light petroleum extract	GC-MS	[13]
**Phytosterols**					
campesterol	*C. pungens* Jan ex Guss	aerial parts	MeOH extract	GC-MS	[6]
9,19-cyclolanostan-3-ol,24-methylene	C. pungens Jan ex Guss	aerial parts	MeOH extract	GC-MS	[6]
cycloartenol	*C. pungens* Jan ex Guss	aerial parts	MeOH extract	GC-MS	[6]
γ-sitosterol	*C. pungens* Jan ex Guss	aerial parts	MeOH extract	GC-MS	[6]
stigmasta-5,22-dien-3-ol	*C. pungens* Jan ex Guss	aerial parts	MeOH extract	GC-MS	[6]
stigmast-7-en-3-ol	*C. pungens* Jan ex Guss	aerial parts	MeOH extract	GC-MS	[6]
**Others**					
N-N’-di-*o*-tolylethylendiamine	*C. sicula* L.	whole plant	MeOH extract	IR, NMR	[39]

^1^ GC-MS, gas chromatography-mass spectrometry. ^2^ GC, gas chromatography. ^3^ NMR, nuclear magnetic resonance. ^4^ IR, infrared. ^5^ MS, mass spectrometry. ^6^ EO, essential oil.

**Table 3:** *Cachrys* spp. investigated biological properties.

Biological Activity	Species	Plant Part	Extract	Model	Ref.
Antioxidant	C. crassiloba *(Boiss.) Meikle*	aerial parts	Hydroalcoholic extract	DPPH	[40]
	*C. cristata* DC.	aerial parts and fruits	MeOH, ethyl acetate, acetone and water extracts	DPPH and ABTS assays	[41]
	*C. libanotis* L.	root	hydroalcoholic extracts and fractions	DPPH, FRAP, ferrous iron chelation and β-carotene bleaching assays	[20]
		aerial parts	MeOH extracts (maceration and Naviglio*^®^*)	DPPH and β-carotene bleaching assays	[5]
	*C. microcarpos* M.Bieb.	leaf	EO and MeOH extract	DPPH assay	[34]
	*C. pungens* Jan ex Guss	aerial parts	MeOH extract and its fractions	DPPH and β-carotene bleaching assays	[6]
	*C. sicula* L.	aerial parts	MeOH extracts (maceration and Naviglio*^®^*)	DPPH and β-carotene bleaching assays	[5]
		leaf	EO	ABTS, DPPH and metal chelating assays	[32]
Antibacterial	*C. cristata* DC.	aerial parts and fruits	MeOH, ethyl acetate, acetone and water extracts	micro-well dilution assay	[41]
		leaf, steam	EO	broth microdilution method	[33]
	*C. libanotis* L.	root	hydroalcoholic extracts and fractions	Agar disc diffusion method	[20]
	*C. microcarpos* M.Bieb.	leaf	EO and MeOH extract	disk diffusion assay, microwell dilution Assay	[34]
		aerial parts	EO	TLC-bioautography assay	[35]
Antifungal	*C. cristata* DC.	steam	EO	broth microdilution method	[33]
	*C. microcarpos* M.Bieb.	leaf	EO	disk diffusion assay, MIC agar dilution assay	[34]
		aerial parts	EO	TLC-bioautography assay	[35]
Anti-inflammatory	*C. libanotis* L.	aerial parts	MeOH extracts (maceration and Naviglio*^®^*)	TNF-α and IL-6 inhibition; NO production inhibition; inhibited the phosphorylation of JAK2 and STAT3 proteins	[9]
	*C. microcarpos* M.Bieb.	-	EtOH extract	carrageenan-induced oedema in rats	[42]
	*C. pungens* Jan ex Guss	aerial parts	MeOH extracts (maceration and Naviglio*^®^*)	TNF-α and IL-6 inhibition; NO production inhibition; induced release of IL-10; inhibited the phosphorylation of JAK2 and STAT3 proteins	[9]
Antiproliferative	*C. microcarpos* M.Bieb.	aerial parts	EtOH and aqueous extracts	MTT test on human prostate cancer cells (PC-3)	[43]
Photocytotoxic	*C. libanotis* L.	aerial parts	MeOH extracts (maceration and Naviglio*^®^*)	UVA- irradiated C32 melanoma cells	[5]
	*C. pungens* Jan ex Guss	aerial parts	MeOH extract and its fractions	UVA- irradiated A375 melanoma cells	[6]
	*C. sicula* L.	aerial parts	MeOH extracts (maceration and Naviglio*^®^*)	UVA- irradiated C32 melanoma cells	[5]
AChE and BChE inhibitory activity	*C. sicula* L.	leaf	EO	ABTS, DPPH and metal chelating assays	[32]
XOR inhibitory activity	*C. libanotis* L.	root	hydroalcoholic extracts and fractions	xanthine/XOR system	[20]
Insecticidal	*C. sicula* L.	stems	hexane extract	insect (*Tribolium castaneum*) growth and/or feeding inhibition	[56]

## Data Availability

Not applicable.
